# Bioactive Secondary Metabolites of Two Chinese Edible Boletes, *Phlebopus portentosus* and *Butyriboletus roseoflavus*

**DOI:** 10.3390/molecules30061197

**Published:** 2025-03-07

**Authors:** Zhixuan Wang, Wei Zhou, Yuhang He, Zeyu Zhao, Yang Cao, Shunzhen Luo, Guangyan Ji, Kaiping Ji, Jing Chen, Jiyang Li, Juan Xiong

**Affiliations:** 1 School of Pharmacy, Fudan University, Shanghai 201203, China; 22211030026@m.fudan.edu.cn (Z.W.); 22111030035@m.fudan.edu.cn (Y.H.); 21111030034@m.fudan.edu.cn (Z.Z.); jyli@fudan.edu.cn (J.L.); 2 Department of Chemistry, Fudan University, Shanghai 200438, China; zhouw@fudan.edu.cn; 3 Jinghong Hongzhen Agricultural Science and Technology Co., Ltd., Jinghong 666100, China; caoyang8352@163.com (Y.C.); 18288002627@163.com (S.L.); jiguangyan93@126.com (G.J.); jkpcnlc@126.com (K.J.); 4 Guizhou Hongzhen Fungus Industry Investment and Development Co., Ltd., Zhenfeng 562200, China; 5 State Key Laboratory of Drug Research, Shanghai Institute of Materia Medica, Chinese Academy of Sciences, Shanghai 201203, China

**Keywords:** edible mushrooms, boletes, *Phlebopus portentosus*, *Butyriboletus roseoflavus*, chemical constituents, bioactivities

## Abstract

This study investigated the phytochemical profiles and bioactivities of two edible boletes from Southwestern China, *Phlebopus portentosus* and *Butyriboletus roseoflavus*. A total of 33 secondary metabolites, comprising 15 alkaloids, 4 pulvinic acid derivative pigments, and 14 ergosterols, were isolated and identified. To our best knowledge, boletesine A (**1**), boletesine B (**2**), and *cis*-xerocomic acid (**16**) were previously undescribed compounds. The new structures were established by extensive spectroscopic methods and chemical calculations. Compound **1** features a hitherto unknown hybrid skeleton formed between a 2-formylpyrrole-alkaloid and a dopacetic acid (DOPAC) via a Michael addition reaction. Bioactivity assays revealed the neuroprotective effects of compounds **18** and **19** against Aβ_25–35_- or H_2_O_2_-induced toxicity. In a cytotoxic assay against a small panel of cancer cell lines, compound **9** exhibited significant activity against HeLa cells (IC_50_ = 10.76 µM), while **33** demonstrated broad-spectrum cytotoxicity against Hela229, SGC7901, PC-3, and BEL7402 cells (IC_50s_ in the range of 20~30 µM). Of particular note is the anti-influenza virus activities against A/H3N2 and B/Victoria strains of compounds **22** and **26** (EC_50_ values ranging from 3.6 to 9.6 µM). Along with these, compound **29** showed a moderate antiviral effect against coxsackievirus B3. These findings underscore the therapeutic potential of the two edible boletes in addressing neurodegenerative diseases, cancer, and viral infections, paving the way for their prospective applications in the development of functional foods and pharmaceuticals.

## 1. Introduction

Globally, the demand for functional foods to prevent or treat diseases is on the rise. More than 1000 medicine–food homologous resources have been recorded globally, finding extensive applications in pharmaceuticals, health supplements, and functional foods [1,2]. In China, the National Health Commission has officially recognized 106 species that serve as both food and traditional medicine [3]. Among these, *Poria cocos* and *Ganoderma lucidum* are notable macrofungi with recognized medicinal and nutritional value. Beyond those species officially documented, many other edible foods may also hold potential medicinal value, requiring further scientific research to clarify their nutritional and bioactive substances and to verify their safety and efficacy.

Edible mushrooms are popular for their taste, low energy content, and high mineral and protein content [4]. Although the medicinal value of edible fungi has been recorded in ancient texts, it was not until the 1950s that researchers began to focus on bioactive substances extracted from mushrooms and their potential in drug development [5]. Studies have shown that extracts from some edible mushrooms exhibit a variety of health benefits, including neuroprotective, immune-enhancing, and anticancer properties [4,5]. Large-sized bolete mushrooms are among the four most renowned edible wild fungi. Botanically, they belong to the order Boletales (Agaricomycetes, Basidiomycota), which is classified into five suborders, 16 families, and approximately 1300 described species and varieties [6,7]. Boletes are found ubiquitously in the temperate and montane forests across the USA, Europe, Siberia, and China [6,7]. China, in particular, is a center for diversity and distribution for boletes, with nearly 500 species reported in recent decades. These species are predominantly found in Yunnan, Sichuan, Guangdong, Guangxi, and other regions [8]. Besides essential amino acids and minerals, boletes also contain various secondary metabolites, including polysaccharides, phenolics (with pulvinic acid derivatives as the predominant group), sterols, alkaloids, and terpenoids. These compounds have been shown to enhance immune function and improve microcirculation [5,9,10].

**Figure 1:**
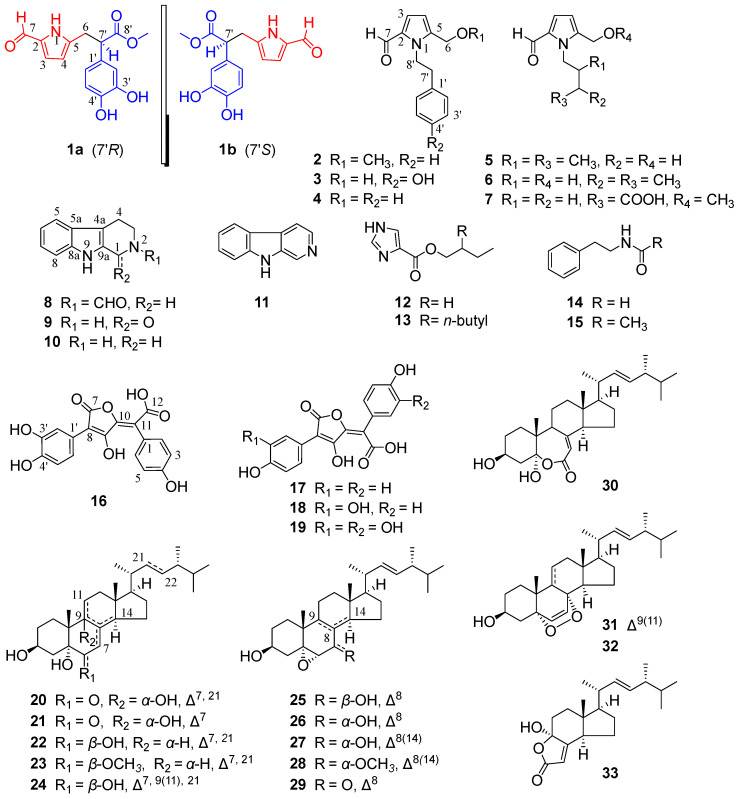
Chemical structures of compounds **1**–**33**. Compounds **8**, **9**, **11**, **16**–**18**, **20**–**29**, and **33** isolated from artificially cultivated *P. portentosus* and compounds **2**–**6** and **8**–**15** isolated from wild *P. portentosus*; compounds **1**, **7**, **17**–**19**, **20**, **22**, **25**–**27**, and **30**–**33** isolated from wild *B. roseoflavus*.

Currently, only two species of boletes have been successfully cultivated [11,12], one of which is *Phlebopus portentosus*. *P. portentosus*, a species belonging to Boletinellaceae (Boletales). It was first artificially cultivated and industrialized by Hongzhen Agricultural Technology Co., Ltd. [12], addressing the issue of insufficient wild resources and enabling the industrialization of functional products. So far, few studies on the secondary metabolites from the fruiting bodies of this macrofungus have been reported, revealing the presence of pyrrole alkaloids and steroids from the Chinese wild *P. portentosus* [13,14,15] and pulvinic acid derivatives from the liquid cultivation of the Thailand *P. portentosus* [16]*. Butyriboletus roseoflavus*, a species from the newly established genus *Butyriboletus* within the Boletaceae family, was recently identified [17]. Previously, only a new ergosterol with anticancer activity was reported from this fungus [18]. The two boletes, *P. portentosus* and *B. roseoflavus*, are popular local mushrooms in Southwest China. However, research on their secondary metabolites remains limited. To fully unlock their potential value—particularly that of cultivated species—this study focuses on investigating the chemical constituents from the fruiting bodies of the two boletes: *P. portentosus* (from both wild and artificial environments) and *B. roseoflavus*. As results, 33 compounds (**1**–**33**, Figure 1) consisting of 15 alkaloids, 4 pulvinic acid derivatives, and 14 ergosterols were isolated and characterized. Herein, their isolation, structural elucidation, and multiple bioactivities including neuroprotective, cytotoxic, and antiviral properties are described.

## 2. Results

### 2.1. Structure Elucidation

**Figure 2:**
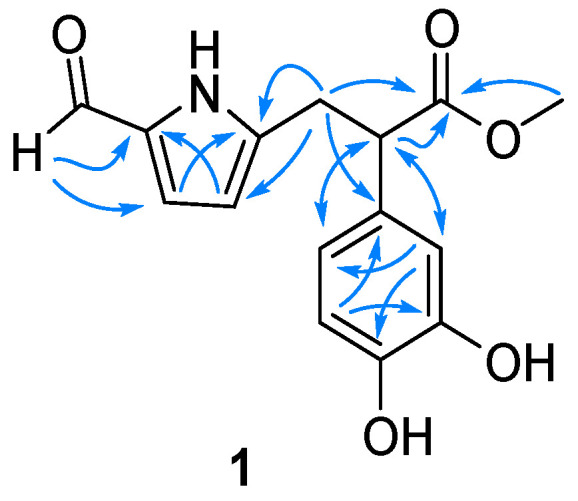
Key HMBC correlations (H → C) of compound **1**.

Compound **1** was isolated as a white amorphous powder. The high-resolution electrospray ionization mass spectrometry (HRESIMS) of **1** showed a sodium adduct ion peak at *m*/*z* 312.0834 [M + Na]^+^ (calcd for C_15_H_15_NO_5_Na, *m*/*z* 312.0842), which in conjunction with the ^13^C NMR spectrum (Table 1) determined a molecular formula of C_15_H_15_NO_5_. This formula indicated that compound **1** is an alkaloid. The ^1^H NMR spectrum displayed signals for an aldehyde proton [*δ*_H_ 9.26 (s, -CHO)], a pair of characteristic aromatic protons of a pyrrole ring [*δ*_H_ 6.87 (d, *J* = 3.9 Hz, H-3), 5.99 (d, *J* = 3.9 Hz, H-4)], an ABX pattern of three aromatic protons [*δ*_H_ 6.74 (d, *J* = 2.2 Hz, H-2′), 6.69 (d, *J* = 8.2 Hz, H-5′), 6.60 (dd, *J* = 8.2, 2.2 Hz, H-6′)] typical for a 1,3,4-trisubtituted phenyl ring, and a methoxy group [*δ*_H_ 3.61 (s, -OCH_3_)] (Table 1). The ^13^C NMR spectrum revealed 15 carbon signals, including two carbonyl carbons (*δ*_C_ 179.9, 175.6), ten aromatic carbons, and three saturated carbons (one methoxy at *δ*_C_ 52.5, one methylene at *δ*_C_ 32.7, and one methyine at *δ*_C_ 52.0). Further analysis of the HMBC spectrum provided insights into the connectivity of the structure (Figure 2). For the pyrrole moiety, the correlations from the formyl proton to C-2/C-3, from H_2_-6 to C-4/C-5, from H-3 to C-5, and from H-4 to C-2 indicated that the formyl and methylene groups were substituted at C-2 and C-5, respectively. Such a substitution pattern is consistent with the co-occurring 2-formylpyrrole alkaloids **3**–**7**.

Meanwhile, a methyl phenylacetate fragment was recognized based on the 1D NMR data (Table 1) and the HMBC correlations from H-7′ to C-1′/C-2′/C-6′/C-8′, from H-2′/H-6′ to C-7′, and from the methoxy protons to C-8′. The observation of the abovementioned ABX coupling system and two oxygen-bearing aromatic carbons [*δ*_C_ 145.9 (C-4′), 146.5 (C-3′)] on the benzene ring, along with the HMBC correlations from H-2′ to C-4′/C-6′ and from H-5′ to C-1′/C-3′, validated the presence of 3′-OH and 4′-OH groups. This 3′,4′-dihydroxyphenylacetic acid (i.e., dopacetic acid, DOPAC) moiety was linked to the pyrrole moiety by the formation of a new C-C bond between C-6 and C-7′, which was evidenced by the diagnostic HMBC correlations from H_2_-6 to C-1′/C-7′/C-8′ and from H-7′ to C-5′/C-6′. Thus, the planar structure of compound **1** was determined as depicted in Figure 1, given a trivial name of boletesine A. A literature survey disclosed that such a hybrid skeleton conjugated by a pyrrole-alkaloid with a DOPAC via a newly formed C-C bond has never been reported until the present study.

**Figure 3:**
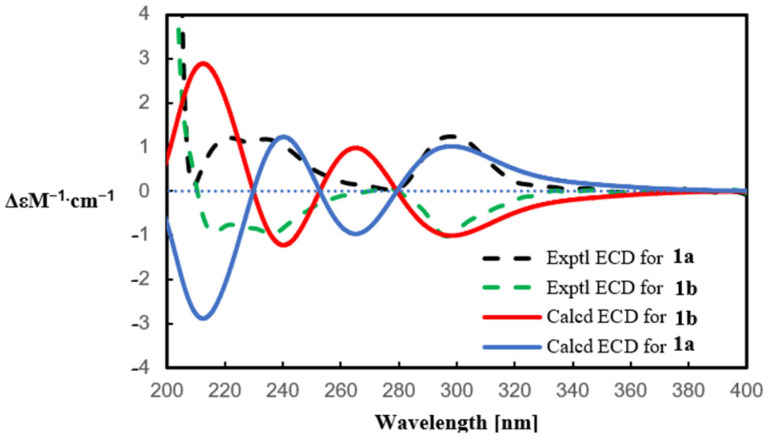
Calculated and experimental ECD curves of **1a** and **1b** in MeOH.

Boletesine A (**1**) was found to be a racemic mixture, since its specific rotation was near-zero. Thereafter, a chiral separation was performed, furnishing a pair of enantiomers, (+)-**1a** and (–)-**1b**. Their absolute configurations were determined by the comparison of the experimental and calculated electronic circular dichroism (ECD) spectra (Figure 3). The positive Cotton effect (CE) at 296 nm in the experimental ECD of (+)-**1a** was consistent with that in the calculated (*R*)-**1a**, while the negative CE at 296 nm of (−)-**1b** was consistent with that in the calculated (*S*)-**1b**. Taken together, the structures of compounds (+)-**1a** and (–)-**1b** were identified as (*R*)-5-[methyl 2-(3,4-dihydroxyphenyl)-propionate]-1*H*-pyrrole-2-carbaldehyde and (*S*)-5-[methyl 2-(3,4-dihydroxyphenyl)-propionate]-1*H*-pyrrole-2-carbaldehyde, respectively.

**Scheme 1:**
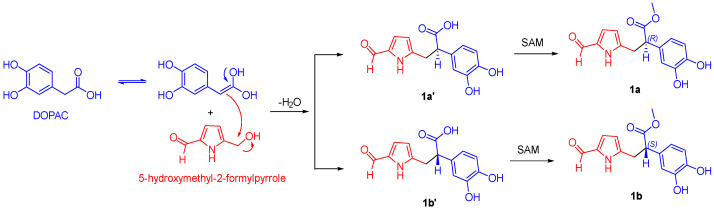
Proposed biosynthetic pathway of compound **1**.

2-Formylpyrrole derivatives have been widely isolated from natural sources, with the non-*N*-substituted 2-formylpyrrole hypothesized to be formed through the condensation of hexosamine [19]. Dopacetic acid (DOPAC), a prevalently distributed natural phenolic acid, serves as the foundation for proposing the biosynthetic pathway of compound **1** (Scheme 1). The hypothesized biosynthesis commences with a Michael addition reaction between DOPAC and 5-hydroxymethyl-2-formylpyrrole. This reaction would give rise to compounds **1a’** and **1b’**, featuring a novel skeleton. The subsequent methylation, presumably mediated by *S*-adenosylmethionine (SAM)-dependent methyltransferases, leads to the formation of compounds **1a** and **1b**. Given that the Michael addition lacks stereoselectivity, compound **1** exists as a pair of enantiomers.

The molecular formula of boletesine B (**2**) was established to be C_15_H_17_NO_2_ based on a protonated ion peak at *m*/*z* 244.1334 [M + H]^+^ (calcd for C_15_H_18_NO_2_, *m*/*z* 244.1332) and the ^13^C NMR data (Table 1). The ^1^H NMR data (Table 1) suggested the presence of an aldehyde proton [*δ*_H_ 9.58 (s, -CHO)], five aromatic proton signals [*δ*_H_ 7.16 (br d, *J* = 7.7 Hz, H-2′/H-6′), 7.29 (br dd, *J* = 7.7, 6.9 Hz, H-3′/H-5′), 7.23 (br t, *J* = 6.9 Hz, H-4′)], a pair of characteristic aromatic protons from the pyrrole ring [*δ*_H_ 6.92 (d, *J* = 4.0 Hz, H-3), 6.19 (d, *J* = 4.0 Hz, H-4)], and a methoxy group [*δ*_H_ 3.31 (s, -OCH_3_)]. These signals suggest that compound **2** features a 2,5-disubstituted pyrrole ring and a mono-substituted benzene ring. The ^13^C NMR spectrum showed 15 carbon signals, comprising one aldehyde carbon (*δ*_C_ 179.4), ten aromatic carbons, three methylenes (one oxygenated at *δ*_C_ 65.6), and one methoxy carbon (*δ*_C_ 57.9). These NMR data were highly compatible with those of the co-isolated inotopyrrole (**4**) [20], with the only difference being the presence of an additional methoxy group in **2**, which was attached to C-6, the only possible substitution site. Consistent with this, the carbon resonance of C-6 in **2** was deshielded by 9.3 ppm when compared with that of **4** (details shown in Table 1). The structure of compound **2**, boletesine B, was thus identified to be 5-(methoxymethyl)-1-phenethyl-1*H*-pyrrole-2-carbaldehyde.

**Figure 4:**
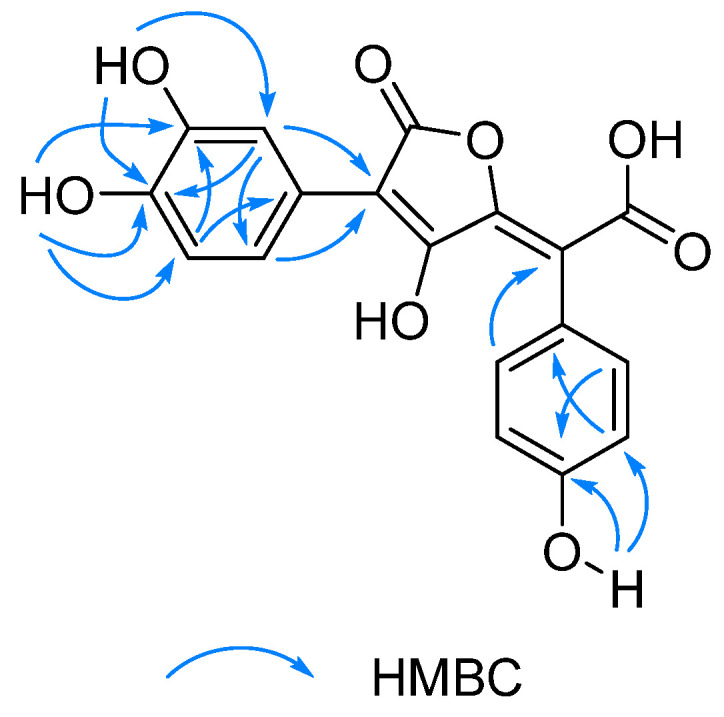
Key HMBC correlations (H → C) of compound **16**.

Compound **16**, isolated as yellow amorphous powder, exhibited a deprotonated molecular ion peak at *m*/*z* 355.0469 [M − H]^−^ (calcd for C_18_H_11_O_8_, *m*/*z* 355.0459) in its HRESIMS, corresponding to a molecular formula of C_18_H_12_O_8_. The ^1^H NMR data of **16** (Table 2) demonstrated the presence of seven aromatic protons, which were assignable to a 1,3,4-trisubstituted benzene ring [ABX system, *δ*_H_ 7.68 (br s, H-2′), 6.67 (d, *J* = 8.5 Hz, H-5′), 7.46 (br d, *J* = 8.5 Hz, H-6′)] and a 1,4-disubstituted benzene ring [AA′BB′ system, *δ*_H_ 6.99 (2H, br d, *J* = 8.4 Hz, H-2/H-6), 6.71 (2H, dd, *J* = 8.4, 2.0 Hz, H-3/H-5)]. Along with these, three phenolic hydroxy proton signals at *δ*_H_ 9.35 (4-OH), 8.78 (3′-OH), and 8.62 (4′-OH)] were also observed. The ^13^C NMR spectrum revealed the presence of 18 carbon signals, consisting of two carboxylic carbons (*δ*_C_ 169.8, 170.2) and 16 olefinic carbons (12 for two benzene rings and the other four arising from two double bonds). These 1D NMR data, along with the UV absorption maxima at 255 nm and 381 nm, suggested that this compound is closely related to those pulvinic acid derivatives, which are responsible for the pigmentation of fruit-bodies belonging to the order Boletales [21]. Detailed analysis of 1D NMR data revealed a high similarity to those of xerocomic acid (**18**) [16,22]. Further analysis of the HMBC correlations (Figure 4) confirmed that compound **16** shares the same planar structure with **18**. Considering the distinctive differences in the 1D NMR data of **16** and **18** in the vicinity of the two acrylic moieties (C-1, C-1′, C-7 to C-12, details shown in Table 2), the double bond between C-10 and C-11 in **16** should adopt a *Z*-configuration rather than the *E*-configuration in **18**. Congruent with this, the C-9 resonance was downfield shifted (*δ*_C_ 169.6 in acetone-*d*_6_) due to anisotropic deshielding by the 12-COOH group in *Z*-**16** compared to the *E*-isomer (*δ*_C_ 160.6). In turn, the C-12 resonance in **16** was upfield shifted by 3.8 ppm. Taken together, compound **16** was determined as the *cis*-isomer of **18**, and given a trivial name of *cis*-xerocomic acid. This *cis*-isomer remained stable for at least two weeks when stored at 4 °C. Eventually, it underwent transformation into the trans-isomer **18**.

By comparing their observed and reported spectroscopic data and physicochemical properties, the known structures (**3**–**15**, **17**–**33**) were identified as pyrrolezanthine (**3**) [23], inotopyrrole (**4**) [20], phlebopine B (**5**) [13], ganoine (**6**) [24], 4-(2-formyl-5-(methoxymethyl)-1*H*-pyrrol-1-yl)butanoic acid (**7**) [25], *N*-formyltryptoline (**8**) [26,27], 1,2,3,4-tetrahydro-1-oxo-β-carboline (**9**) [28], 1,2,3,4-tetrahydro-β-carboline (**10**) [29], β-carboline (**11**) [30], butyl 1*H*-imidazole-4-carboxylate (**12**) [31], 2-ethylhexyl 1*H*-imidazole-4-carboxylate (**13**) [32], *N*-phenethylformamide (**14**) [33], *N*-phenethylacetamide (**15**) [34], atromentic acid (**17**) [35], xerocomic acid (**18**) [16,25], variegatic acid (**19**) [36], 3β,5*a*,9α-trihydroxy-ergosta-7,22-dien-6-one (**20**) [37], (24*S*)-3β,5α,9α-trihydroxyergosta-7-en-6-one (**21**) [38], cerevisterol (**22**) [39], (22*E*,24*R*)-3β,5α-dihydroxy-6β-methoxy-ergosta-7,22-diene (**23**) [37], (22*E*,24*R*)-ergosta-7,9(11),22-trien-3β,5α,6β-triol (**24**) [40], (22*E*,24*R*)-5α,6α-epoxy-ergosta-8,22-dien-3β,7β-diol (**25**) [41], (22*E*,24*R*)-5α,6α-epoxy-ergosta-8,22-dien-3β,7α-diol (**26**) [42], (22*E*,24*R*)-5α,6α-epoxy-ergosta-8(14),22-dien-3β,7α-diol (**27**) [43], (22*E*,24*R*)-5α,6α-epoxy-7α-methoxy-ergosta-8(14),22-dien-3β-ol (**28**) [44], (22*E*,24*R*)-5α,6α-epoxy-3β-hydroxy-ergosta-8,22-dien-7-one (**29**) [45], (22*E*,24*R*)-3β,5α-dihydroxy-ergosta-7,22-dien-6,5-olide (**30**) [46], 9,11-dehydroergosterol peroxide (**31**) [47], (22*E*,24*R*)-5α,8α-epidioxyergosta-6,22-dien-3-ol (**32**) [48], and demethylincisterol A_3_ (**33**) [49], respectively. Notably, the ^1^H and ^13^C NMR spectra (in CD_3_OD) of compound **8** showed two sets of similar signals, in a ratio of 3:1 (**8a**:**8b**). This rotameric behavior (atropisomerism), which should result from the substitution of the formyl group at N_2_, has never been reported until the present study. The fully assigned 1D NMR data for the two atropisomers of **8** were reported for the first time (see Section 3.4).

Structurally, the isolates could be categorized as three groups: alkaloids (**1**–**15**), pulvinic acid-derived pigments (**16**–**19**), and ergosterols (**20**–**33**). Among them, compounds **1**, **7**, **17**–**19**, **20**, **22**, **25**–**27**, and **30**–**33** were isolated from B. roseoflavus and all of them were reported from this species for the first time [18]. Compounds **8**, **9**, **11**, **16**–**18**, **20**–**29**, and **33** were isolated from the artificially cultivated *P. portentosus*, whereas compounds **2**–**6** and **8**–**15** were isolated from the wild *P. portentosus* guided by the HPLC-PDA-based dereplication strategy; most of these isolates (with the exception of **4**–**6**, **17**, **18**, and **22** [13,14,15,16]) were reported for the first time from *P. portentosus*.

As can be seen above, all the three groups of natural products were found in both the edible boletes. A noticeable difference is that, the alkaloid content and diversity in *P. portentosus* were higher than those found in *B. roseoflavus*. The 2-formylpyrrole alkaloids exist in both two boletes, but the β-carboline-type alkaloids are only isolated from the *P. portentosus* (either wild or cultivated species). In addition, comparative metabolomic analysis between wild and cultivated *P. portentosus* revealed no significant overall differences in secondary metabolite composition. Both forms exhibited substantial accumulation of long-chain fatty acids, sterols, alkaloids, and pulvinic acid derivative pigments, suggesting that artificial cultivation preserves the fundamental metabolic characteristics of this species. Notably, while maintaining the core metabolic profile, cultivated specimens demonstrated enhanced alkaloid diversity through the emergence of novel pyrrole-type alkaloids in addition to the conserved β-carboline alkaloid framework. These findings collectively indicate the biological feasibility of artificial cultivation for metabolite production while highlighting subtle metabolic variations between cultivation methods.

### 2.2. Biological Activities

In order to explore the medicinal potential of the two edible boletes, extensive activity screening has been conducted on isolated compounds.

#### 2.2.1. Neuroprotective Activity

Neurodegenerative diseases (NDs) are chronic, irreversible, and progressive disorders caused by neuronal degeneration. These conditions result in progressive cognitive decline due to selective neuronal dysfunction, synaptic loss, and neuronal cell death, with Alzheimer’s disease (AD) and Parkinson’s disease (PD) being prominent examples [50]. Therefore, the search for neuroprotective compounds holds promise for discovering potential drugs to prevent or treat NDs. To begin with, inspired by the neuroprotective and anti-neuroinflammatory activities of alkaloid and phenolic compounds [13,14,15], compounds **1**–**19** were evaluated for their protective action against the neuro-injury. Neuroprotective effects against Aβ_25−35_- or H_2_O_2_-induced neurotoxicity were assessed in SH-SY5Y cells.

**Figure 5:**
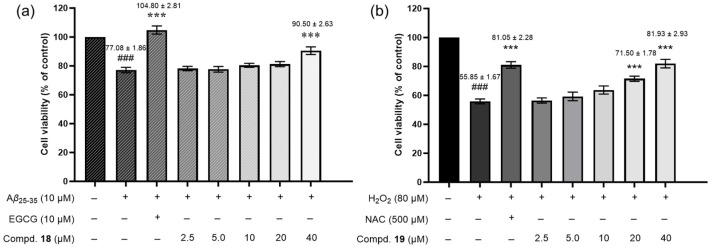
Neuroprotective activity of compounds **18** (**a**) and **19** (**b**) in SH-SY5Y cells. The cell viability was assessed by measuring the MTT reduction. Epigallocatechin gallate (EGCG) and *N*-acetylcysteine (NAC) were positive controls. Four independent experiments were carried out in triplicate. The data are expressed as a percentage of control value and are represented by means ± SEM. ^###^ *p* < 0.00l vs. control group, *** *p* < 0.001 vs. Aβ_25–35_ group (**a**) or *** *p* < 0.001 vs. H_2_O_2_ group (**b**).

As illustrated in Figure 5a, the results showed that Aβ_25−35_ reduced cell viability to 77.08%. In contrast, treatment with compound **18** (40 μM) significantly increased cell viability to 90.50%, though this effect was slightly weaker than the positive control EGCG, which restored viability to 104.80% at 10 µM. Compared to the Aβ_25−35_ model group, compound **18** at 40 μM significantly enhanced cell survival. In the H_2_O_2_-induced neurotoxicity assay (Figure 5b and Table S2), compound **19** was most effective. It increased cell viability significantly to 71.50% at 20 µM and 81.93% at 40 µM, outperforming the positive control NAC, which increased viability to only 81.05% at 500 µM. Other compounds displayed weak activities, with a <10% increase in cell survival over the Aβ_25−35_ or H_2_O_2_ group at 20 µM.

#### 2.2.2. Cytotoxicity Activity

Given that the ergosterols and pulvinic acid derivatives were previously reported to demonstrate anti-cancer activities [8,14], most of the isolates were tested for their cytotoxicities against a small panel of human cancer cell lines (Hela229, SGC7901, PC-3, BEL7402, and MCF-7/ADR). The results (details shown in Tables S3 and S4) revealed that only a β-carboline alkaloid (**9**) and an ergosterol (**33**) exhibited remarkable activities. Compound **9** showed cytotoxic effects against Hela229 and BEL7402 cells, with IC_50_ values of 10.76 and 41.54 µM, respectively. Compound **33** showed a similar cytotoxic profile against Hela229, SGC7901, PC-3, and BEL7402 cell lines, with IC_50_ values of 24.45, 26.66, 28.43, and 27.72 µM, respectively. The other compounds were deemed inactive (IC_50_ > 50 µM). Doxorubicin was used as a positive control, with IC_50_s ranging from 0.75 to 35.2 µM.

#### 2.2.3. Antiviral Activity

**Figure 6:**
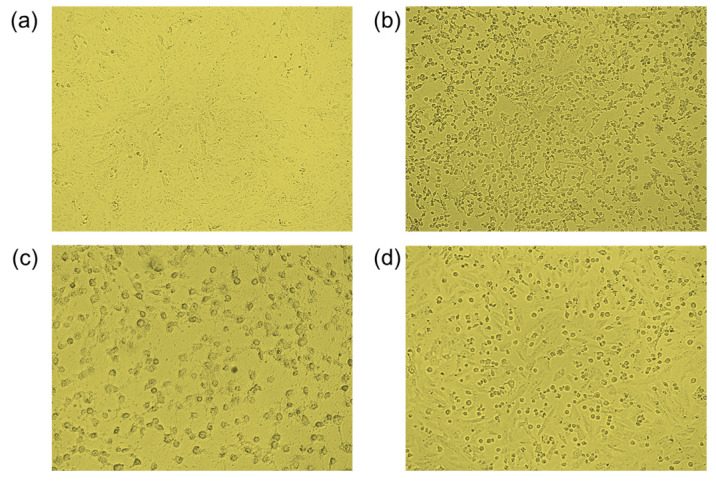
Morphological assessment of compound **29** against CVB in vitro (magnification: 100×). CVB-infected Vero cells were treated with 100 μg/mL of compound **29** and ribavirin (positive control). Cell morphology was assessed by microscope. (**a**) CVB-uninfected Vero cells. (**b**) CVB-infected Vero cells. (**c**) CVB-infected Vero cells treated with ribavirin. (**d**) CVB-infected Vero cells treated with compound **29**.

**Figure 7:**
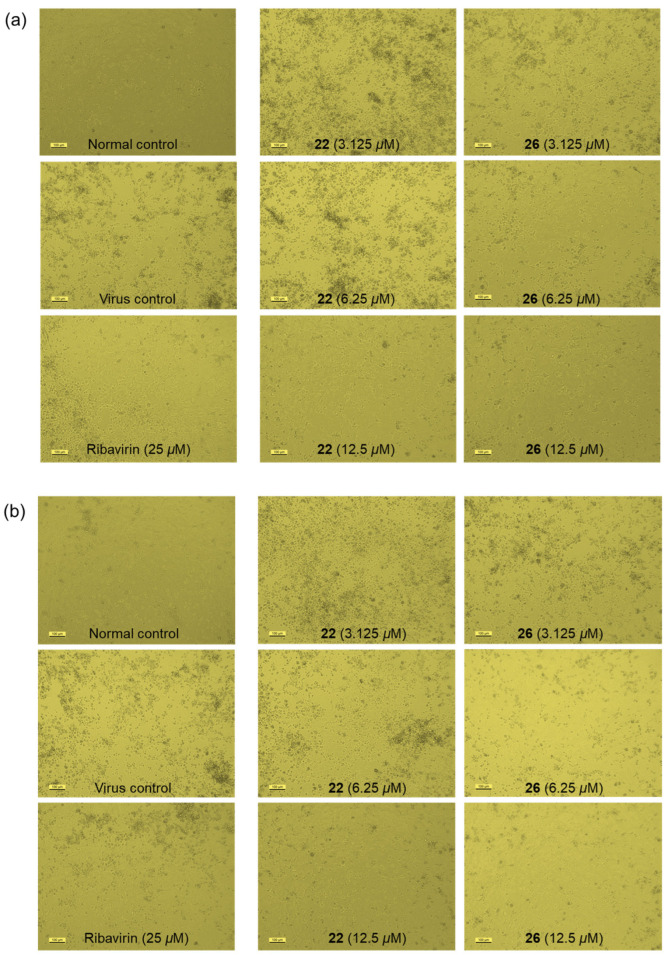
Cytopathic effect (CPE) images of MDCK cells. Ribavirin was used as a positive control. (**a**) CPE images of compounds **22** and **26** against influenza virus A/H3N2 strain. (**b**) CPE images of compounds **22** and **26** against influenza virus B/Victoria strain.

The antiviral activities of most isolates on coxsackievirus and influenza virus were screened by the cytopathic effect (CPE) inhibition assay [51,52]. In the anti-coxsackievirus assay, compound **29** (100 μg/mL) reversed the cytopathic morphology of Vero cells severely infected with coxsackievirus B3 (CVB), and increased cell survival by 35.4% (Figure 6). The anti-influenza effect was evaluated against influenza virus A (H3N2 strain) and B (Victoria strain) in MDCK cells. As shown in Figure 7 and Table S5, compounds **22** and **26** exhibited stronger anti-influenza activity than the positive control ribavirin, with respective EC_50_ values of 9.6 μM/6.6 μM (**22**) and 4.0 μM/3.6 μM (**26**). Ribavirin showed EC_50_ values of 28.3 μM against influenza A and 20.6 μM against influenza B. Congruent with our findings, several highly conjugated ergosterols from a marine-derived fungus were previously reported to show a similar anti-influenza activity [53].

Anti-influenza virus activity involves multiple targets, including neuraminidase (NA), M2 ion channel blockers, hemagglutinin (HA), viral polymerase complex, and viral nucleoprotein [54,55]. Molecular docking experiments revealed that β-sitosterol, which shares structural similarities with steroids **22** and **26**, exhibited the highest binding affinity to the head domain of HA [56]. This suggested that the antiviral activity of compounds **22** and **26** may also arise from their interaction with HA, potentially blocking viral fusion with the host cell membrane, thereby preventing the release of viral genetic material and subsequent infection. However, the precise mechanism requires further investigation.

## 3. Materials and Methods

### 3.1. General Experimental Procedures

Optical rotations were measured on a Rudolf Autopol IV automatic polarimeter (Rudolph Research Analytical, Hackettstown, NJ, USA). High-resolution electrospray ionization mass spectra (HRESIMS) were acquired using an AB SCIEX Triple TOF 5600 spectrometer (Framingham, MA, USA). Ultraviolet (UV) and infrared (IR) spectra were recorded on Hitachi U-2900E (Tokyo, Japan) and Avatar 360 ESP FTIR spectrometers (Thermo Fisher Scientific, Waltham, MA, USA), respectively. Electronic circular dichroism (ECD) data were obtained using a JASCO-810 spectropolarimeter (Hachioji, Japan). Nuclear magnetic resonance (NMR) spectra were recorded on a Bruker Avance III 400 or 600 MHz spectrometer (Billerica, MA, USA). Chemical shifts are expressed in parts per million (*δ* scale) and referenced to the residual solvent signals. Semi-preparative high-performance liquid chromatography (HPLC) was conducted on a Shimadzu LC-20AT system (Kyoto, Japan) with an SPD-M20A prominence diode array (PDA) detector, using the Cosmosil cholester packed column (Kyoto, Japan, 10 mm × 250 mm, 5 μm), Waters X-Bridge ODS column (Milford, MA, USA, 250 × 10 mm, 5 μm), or Agilent Zorbax SB-Phenyl column (Santa Clara, CA, USA, 250 × 9.4 mm, 5 μm). Silica gel (100–200 mesh, Qingdao Haiyang Chemical Co., Ltd., Qingdao, China), CHP20P MCI gel (75–150 μm, Mitsubishi Chemical Corporation, Kyoto, Japan), and Sephadex LH-20 gel (GE Healthcare Bio-Sciences AB, Chicago, IL, USA) were used for column chromatography (CC). Gel-precoated plates (GF254, 0.2 mm, Yantai Jiangyou Silica Gel Development Co., Ltd., Yantai, China) were used for thin-layer chromatography (TLC) detection, with spots being visualized under UV light (254 and/or 365 nm) and by spraying with vanillin in 10% (*v*/*v*) H_2_SO_4_/EtOH reagent or Dragendorff’s reagent.

### 3.2. Mushroom Materials

The artificially cultivated *Phlebopus portentosus* fruiting bodies (fresh sample) were harvested in November 2022 from the Xishuangbanna base of Hongzhen Biotechnology Development (Group) Co., Ltd. in Jinghong, China. Wild *Phlebopus portentosus* fruiting bodies (fresh sample) were collected in December 2022 from Jinghong, Xishuangbanna, Yunnan Province, and identified by one of the authors (Y.C.). Wild *Butyriboletus roseoflavus* fruiting bodies (air-dried mushroom in slice, 4.5 kg) were purchased by one of the authors (Y.C.) from the local market in Jinghong in December 2023, and identified by Prof. Gang Wu, Kunming Institute of Botany, Chinese Academy of Sciences. All the specimens (No. 20221101 for the cultivated *P. portentosus*, 20221201 for the wild *P. portentosus*, and 20231201 for *B. roseoflavus*) were deposited at the Department of Natural Medicine, School of Pharmacy, Fudan University.

### 3.3. Extraction and Isolation

#### 3.3.1. Extraction and Isolation of Cultivated *P. portentosus*

The artificially cultivated fresh mushroom (14.6 kg) was cut into small pieces and placed in a bucket, to which 80% MeOH (10.0 L) was added in portions and mixed well. The mixture was macerated five times, then the extract was concentrated under reduced pressure and evaporated to dryness to obtain 831.5 g of brown crude extract. The total extract was dispersed in 3% tartaric acid aqueous solution, then ethyl acetate (EtOAc) was added (equal volume) for extraction five times to obtain 18.8 g of EtOAc-soluble portion. The aqueous layer was adjusted to pH 9–10 with Na_2_CO_3_ and then extracted five times with an equal volume of chloroform, resulting in the crude alkaloid fraction (8.6 g). The aqueous layer was further extracted with *n*-butanol (*n*-BuOH) five times, yielding 20.9 g of *n*-BuOH-soluble portion.

The EtOAc extract (18.8 g) was subjected to coarse separation using MCI column chromatography, eluted with a gradient of MeOH-H_2_O (from 20% to 100% MeOH, *v/v*), and 15 fractions (Fr A1~Fr A15) were obtained. Fr A5 (450 mg) was further separated by Sephadex LH-20 (MeOH), yielding 12 subfractions (Fr A5-1 to Fr A5-12). Fr A5-10 (15.0 mg) was identified as compound **16**. Fr A5-9 and Fr A5-11 were further purified by semi-preparative HPLC [column: Zorbax SB-Phenyl; mobile phase: MeCN-H_2_O (containing 0.05% FA) 30:70, *v*/*v*] to yield compound **17** (1.8 mg, *t*_R_ = 11.3 min) and compound **18** (1.7 mg, *t*_R_ = 5.41 min), respectively. Fr A13 (300 mg) was separated by Sephadex LH-20 (MeOH-CH_2_Cl_2_, 50:50, *v*/*v*) into six subfractions (Fr A13-1 to Fr A13-6). Compound **33** [1.2 mg, *t*_R_ = 14.5 min; column: X-Bridge C18, mobile phase: MeCN-H_2_O (0.05% FA, 85:15, *v*/*v*)] was obtained from Fr A15-5 via semi-preparative HPLC.

Fr A14 (2.9 g) was subjected to silica gel CC with a gradient elution system of CH_2_Cl_2_: MeOH (99:1 → 5:1), resulting in 17 subfractions (Fr A14-1 to Fr A14-17). Fr A14-7 (30 mg) was purified by semi-preparative HPLC (column: Cosmosil cholester, mobile phase: MeOH-H_2_O, 90:10, *v*/*v*) to obtain compound **24** (2.0 mg, *t*_R_ = 13.6 min). Fr A14-9 (30 mg) was purified by semi-preparative HPLC (column: Cosmosil cholester; mobile phase: MeOH-H_2_O, 95:10, *v*/*v*) to obtain compound **23** (4.5 mg, *t*_R_ = 13.0 min). Compounds **20** (6.0 mg, *t*_R_ = 21.8 min) and **25** (1.3 mg, *t*_R_ = 24.7 min) were isolated from Fr A14-10 by semi-preparative HPLC (column: X-Bridge C18; mobile phase: MeCN-H_2_O, 87:13, *v*/*v*); the remaining fractions were pooled and purified by semi-preparative HPLC (column: Cosmosil cholester; mobile phase: MeOH-H_2_O, 95:5, *v*/*v*) to yield compounds **21** (1.6 mg, *t*_R_ = 18.4 min), **27** (3.5 mg, *t*_R_ = 13.1 min), and **26** (1.0 mg, *t*_R_ = 13.7 min). Fr A14-11 (250 mg) was subjected to ODS column chromatography (from 20% to 100% MeOH in H_2_O, *v*/*v*) and then semi-preparative HPLC to obtain compound **28** [1.5 mg, *t*_R_ = 17.9 min; column: Zorbax SB-Phenyl; mobile phase: MeOH-H_2_O (0.05% FA), 88:12, *v*/*v*]. Fr A14-12 (65 mg) was purified by semi-preparative HPLC [column: X-Bridge C18; mobile phase: MeCN-H_2_O (0.05% FA), 83:17, *v*/*v*] to obtain compound **22** (6.0 mg, *t*_R_ = 25.0 min). Fr A15 (1.6 g) was separated by silica gel CC with a gradient elution system of CH_2_Cl_2_: MeOH (99:1 → 5:1), resulting in 12 subfractions (Fr A15-1 to Fr A15-12). Fr A15-4 (17 mg) was purified by semi-preparative HPLC (column: X-Bridge C18; mobile phase: MeCN-H_2_O, 85:15, *v*/*v*) to obtain compound **29** (3.0 mg, *t*_R_ = 14.5 min).

The CHCl_3_-soluble alkaloid extract (8.9 g) was fractionated by CC over MCI gel, eluted with MeOH-H_2_O (20% → 100%, *v*/*v*) to obtain 12 fractions (Fr A16 to Fr A27). Fr A22 was purified by semi-preparative HPLC (column: X-Bridge C18; mobile phase: MeCN-H_2_O, 35:65, *v*/*v*) to furnish compounds **9** (2.2 mg, *t*_R_ = 12.3 min) and **8** (1.9 mg, *t*_R_ = 15.5 min). Compound **11** (5.7 mg, *t*_R_ = 16.1 min) was obtained from Fr A23 by semi-preparative HPLC (MeOH-H_2_O, 62:38, *v*/*v*; column: X-Bridge C18). For the above HPLC conditions, 0.05% (*v*/*v*) diethylamine (DEA) was added to the aqueous phase to improve peak shape and separation efficiency.

#### 3.3.2. Extraction and Isolation of Wild *P. portentosus*

The wild fresh mushroom (4.0 kg) was chopped and extracted in batches with 80% MeOH (5.0 L) at room temperature five times. The extract was concentrated under reduced pressure and evaporated to dryness to obtain 244 g of brown crude extract. The total extract was dispersed in 3% tartaric acid aqueous solution, then extracted five times with EtOAc, yielding 8.63 g of EtOAc extract. The aqueous layer was adjusted to pH 9–10 with Na_2_CO_3_, then extracted with CHCl_3_ to obtain the alkaloid fraction (1.65 g).

The non-alkaloid EtOAC extract was fractionated into nine subfractions by CC over MCI gel. However, it was discovered that these subfractions either contained ergosterols having the same retention time as those isolated from the cultivated mushroom, or were abundant in long-chain fatty acids. In contrast, analyses of the subfractions obtained from the alkaloid CHCl_3_ extract revealed the wild mushroom harbored a significantly greater variety of structurally diverse alkaloids compared to the cultivated mushroom. The separation process of this portion is described in detail as follows.

The CHCl_3_ extract (1.65 g) was chromatographed on MCI gel column using gradient solvents of MeOH-H_2_O (20% → 100%, *v*/*v*), resulting in nine subfractions (Fr B1 to Fr B9). Fr B4 (39 mg) was purified by semi-preparative HPLC (column: Zorbax SB-Phenyl, mobile phase: MeCN-H_2_O 25:75, *v*/*v*) to afford compounds **14** (3.1 mg, *t*_R_ = 13.8 min), **15** (2.7 mg, *t*_R_ = 14.9 min), and **3** (2.1 mg, *t*_R_ = 17.6 min). Fr B5 (108 mg) was divided into six subfractions (Fr B5-1 to Fr B5-6) by CC over Sephadex LH-20 (MeOH). Fr B5-1 was purified by semi-preparative HPLC (column: Zorbax SB-Phenyl; mobile phase: MeOH-H_2_O, 91:9, *v*/*v*) to obtain compound **13** (2.3 mg, *t*_R_ = 8.9 min). Fr B5-2 was also purified by semi-preparative HPLC (column: Zorbax SB-Phenyl; mobile phase: MeOH-H_2_O, 57:43, *v*/*v*) to yield compounds **5** (1.8 mg, *t*_R_ = 12.1 min) and **6** (2.4 mg, *t*_R_ = 13.0 min). Fr B6 (74 mg) was chromatographed on an ODS column (MeOH-H_2_O, 50% → 100%, *v*/*v*) to obtain four subfractions (Fr B6-1 to Fr B6-4). Fr B6-2 was separated by semi-preparative HPLC (column: X-Bridge C18; mobile phase: MeCN-H_2_O, 35:65, *v*/*v*) to yield compounds **9** (2.6 mg, *t*_R_ = 10.1 min), **8** (2.3 mg, *t*_R_ = 12.6 min), and **4** (2.1 mg, *t*_R_ = 18.6 min) in turn. Compound **11** (1.9 mg, *t*_R_ = 21.0 min) was purified from Fr B6-3 by semi-preparative HPLC (column: Zorbax SB-Phenyl; mobile phase: MeCN-H_2_O, 43:57, *v*/*v*). Fr B8 (370 mg) was isolated by Sephadex LH-20 (MeOH) and semi-preparative HPLC to afford compound **10** [2.6 mg, *t*_R_ = 6.4 min; column: X-Bridge C18; mobile phase: MeOH-H_2_O (0.05% DEA), 75:25, *v*/*v*)] and compound **2** [3.3 mg, *t*_R_ = 11.8 min; column: Zorbax SB-Phenyl; mobile phase: MeOH-H_2_O, 72:28, *v*/*v*]. Fr B9 (101 mg) was also separated by Sephadex LH-20 (MeOH) and further purified by semi-preparative HPLC (column: Zorbax SB-Phenyl; mobile phase: MeCN-H_2_O, 78:22, *v*/*v*) to obtain compound **12** (2.3 mg, *t*_R_ = 10.5 min).

#### 3.3.3. Extraction and Isolation of *B. roseoflavus*

The dried fruiting bodies (4.50 kg) of *B. roseoflavus* were chopped and extracted with 80% MeOH (4.0 L) at room temperature five times. The extract was concentrated under reduced pressure and evaporated to dryness to obtain 1500 g of brown crude extract. Upon completion of the acid extraction and alkaline precipitation pretests, the TLC and HPLC detection indicated an extremely low alkaloid content. Consequently, in the ensuing experiment, the conventional extraction method was adopted. The total extract was dispersed in water, and then sequentially extracted with petroleum ether (PE) and EtOAC, yielding PE extract (120 g) and EtOAc extract (15 g).

The EtOAc extract (15.0 g) was subjected to CC over an MCI column and eluted with a MeOH-H_2_O (20% → 100%, *v*/*v*) gradient to provide 14 subfractions (Fr C1 to Fr C14). Subfraction Fr C3 (400 mg) was separated by Sephadex LH-20 (MeOH) into eight subfractions (Fr C3-1 to Fr C3-8). Fr C3-8 was identified as compound **19** (20.0 mg). Fr C3-4 was further purified by semi-preparative HPLC [mobile phase: MeCN-H_2_O (0.05% FA), 0–5 min: 20%; 5–45 min: 20–90%, *v*/*v*] to obtain compound **1** (2.0 mg, *t*_R_ = 19.60 min). In this part, all the HPLC purifications were carried out on the Zorbax SB-Phenyl column. Fr C6 (340 mg) was separated by silica gel column chromatography and eluted with a gradient of CH_2_Cl_2_: MeOH (0.1% FA) (99:1 → 1:1) to yield nine subfractions (Fr C6-1 to Fr C6-9). Fr C6-4 was purified by semi-preparative HPLC [mobile phase: MeCN-H_2_O (0.05% FA), 30:70, *v*/*v*] to yield compound **7** (2.1 mg, *t*_R_ = 9.9 min). In a similar way, separation of Fr C6-7 on the same HPLC column [mobile phase: MeCN-H_2_O (0.05% FA), 27:73, *v*/*v*] afforded compounds **18** (5.8 mg, *t*_R_ = 7.9 min) and **17** (4.0 mg, *t*_R_ = 15.1 min). Fr C14 (240 mg) was divided into four subfractions (Fr C14-1 to Fr C14-4) by Sephadex LH-20 (MeOH). Fr C14-3 was further purified by semi-preparative HPLC [mobile phase: MeCN-H_2_O (0.05% FA), 72:28, *v*/*v*] to obtain compounds **31** (2.3 mg, *t*_R_ = 16.3 min) and **32** (1.7 mg, *t*_R_ = 17.5 min).

The PE extract (120 g) was segmented via silica gel column chromatography with a gradient elution of PE/EtOAc (50:1 → 1:20 → neat EtOAc) to obtain seven subfractions (Fr C15~Fr C21). Fr C18 (2.13 g) was further purified by semi-preparative HPLC mobile phase: MeCN-H_2_O (0.05% FA), 70:30, *v*/*v*] to obtain compound **33** (2.5 mg, *t*_R_ = 10.4 min). Fr C20 (1.51 g) was further separated by CC over silica gel (CH_2_Cl_2_: MeOH, 99:1 → 1:1) to yield nine subfractions (Fr C20-1 to Fr C20-9). Further purification of Fr C20-6 by semi-preparative HPLC [mobile phase: MeCN-H_2_O (0.05% FA), 63:37, *v*/*v*] gave compounds **26** (10.1 mg, *t*_R_ = 16.5 min) and **27** (5.1 mg, *t*_R_ = 18.2 min). Compound **22** (5.0 mg, *t*_R_ = 16.5 min) was purified from Fr C20-7 by semi-preparative HPLC [mobile phase: MeCN-H_2_O (0.05% FA), 63:37, *v*/*v*]. Fr C20-8 (220 mg) was fractioned by Sephadex LH-20 (MeOH) to obtain five subfractions (Fr C20-8a to Fr C20-8e). Compounds **20** (1.2 mg, *t*_R_ = 15.0 min) and **25** (1.8 mg, *t*_R_ = 16.1 min) were obtained from Fr C20-8c by semi-preparative HPLC [mobile phase: MeCN-H_2_O (0.05% FA), 62:38, *v*/*v*]. Fr C20-8d was separated by semi-preparative HPLC [MeOH-H_2_O (0.05% FA), 85:15, *v*/*v*] to afford compound **30** (1.2 mg, *t*_R_ = 16.2 min).

In line with the descriptions above, flowcharts presenting the general separation process of compounds are provided in the Supplementary Materials (Figures S1–S3) for easier visualization. The purity of all the above compounds has been confirmed by HPLC and ^1^H NMR analysis, with each compound exhibiting a purity greater than 95%.

#### 3.3.4. Chiral Separation

Chiral separation of compound **1** was conducted under normal phase HPLC conditions using a CHIRALPAK IC column (4.6 mm I.D. × 250 mm L, 5 μm, Daicel Chemical Industries, Tokyo, Japan) and a Waters e2695 system with a 2998 prominence diode array (PDA). The elution solvent was hexane/isopropanol (67:33); flow rate: 1.0 mL/min; column temperature: 30 °C. The separation resulted in two enantiomers, (+)-**1a** (*t*_R_ = 11.40 min) and (–)-**1b** (*t*_R_ = 13.30 min).

### 3.4. Spectroscopic Data of the Isolated Compounds

(*R*)-Boletesine A [(+)-**1a**)]: amorphous powder $[\alpha {]}_{D}^{20}$ +50.0 (*c* 0.10, MeOH); UV (MeOH) λmax (log ε) 297 (2.58), 204 (2.83) nm; ECD (c 1.15 × 10^−3^ M, MeOH) λ_max_ (Δε) 295 (+1.2), 233 (+1.2), 221 (+1.2) nm; ^1^H and ^13^C NMR data see Table 1; (+) HRESIMS *m*/*z* 312.0834 [M + Na]^+^ (calcd for C_15_H_15_NO_5_Na, *m*/*z* 312.0842).

(*S*)-Boletesine A [(–)-**1b**]: amorphous powder; $[\alpha {]}_{D}^{20}$ –43.6 (*c* 0.10, MeOH); UV (MeOH) λmax (log ε) 297 (2.58), 204 (2.83) nm; ECD (c 1.15 × 10^−3^ M, MeOH) λ_max_ (Δε) 296 (−1.0), 236 (−1.0), 216 (−0.9) nm; ^1^H and ^13^C NMR data see Table 1; (+) HRESIMS *m*/*z* 312.0834 [M + Na]^+^ (calcd for C_15_H_15_NO_5_Na, *m*/*z* 312.0842).

Boletesine B [=5-(methoxymethyl)-1-phenethyl-1*H*-pyrrole-2-carbaldehyde, **2**]: white, amorphous powder; UV (MeOH) λ_max_ (log ε) 292 (3.68), 204 (3.65) nm;^1^H and ^13^C NMR data see Table 1; HRESIMS *m*/*z* 244.1334 [M + H]^+^ (calcd for C_15_H_18_NO_2_, *m*/*z* 244.1332).

*N_2_*-Formyl-1,2,3,4-tetrahydro-β-carboline (**8**): brown, amorphous powder; ^1^H NMR (CD_3_OD, 600 MHz) data of **8a**: *δ*_H_ 8.25 (1H, br s, -CHO), 7.40 (1H, br d, *J* = 7.7 Hz, H-5), 7.29 (1H, br d, *J* = 8.0 Hz, H-8), 7.07 (1H, br dd, *J* = 8.0, 7.3 Hz, H-7), 6.99 (1H, br dd, *J* = 7.7, 7.3 Hz, H-6), 4.71 (2H, br s, H-1), 3.81 (2H, t, *J* = 5.8 Hz, H-3), 2.86 (2H, br t, *J* = 5.8 Hz, H-4); ^1^H NMR (CD_3_OD, 600 MHz) data of **8b**: *δ*_H_ 8.30 (1H, br s, -CHO), 7.40 (1H, br d, *J* = 7.7 Hz, H-5), 7.29 (1H, br d, *J* = 8.0 Hz, H-8), 7.07 (1H, br dd, *J* = 8.0, 7.3 Hz, H-7), 6.99 (1H, br dd, *J* = 7.7, 7.3 Hz, H-6), 4.68 (2H, br s, H-1), 3.90 (2H, t, *J* = 5.8 Hz, H-3), 2.80 (2H, br t, *J* = 5.8 Hz, H-4)]; ^13^C NMR (CD_3_OD, 125 MHz) data of **8a**: *δ*_C_ 164.4 (-CHO), 138.2 (C-8a), 130.0 (C-9a), 128.2 (C-5a), 122.4 (C-7), 119.4(C-6), 118.6 (C-5), 111.9 (C-8), 108.1 (C-4a), 45.6 (C-3), 39.4 (C-1), 23.1 (C-4); ^13^C NMR (CD_3_OD, 125 MHz) data of **8b**: *δ*_C_ 164.0 (-CHO), 138.1 (C-8a), 130.6 (C-9a), 128.2 (C-5a), 122.4 (C-7), 120.0 (C-6), 118.6 (C-5), 111.9 (C-8), 108.6 (C-4a), 45.0 (C-3), 39.7 (C-1), 21.8 (C-4)]; HRESIMS *m*/*z* 201.1021 [M + H]^+^ (calcd for C_12_H_12_N_2_O, *m*/*z* 201.1022).

*cis*-Xerocomic acid [=(*Z*)-2-(4-(3,4-dihydroxyphenyl)-3-hydroxy-5-oxofuran-2(5*H*)-ylidene)-2-(4-hydroxyphenyl) acetic acid, **16**]: yellow, amorphous powder; UV (MeOH) λ_max_ (log ε) 381 (3.88), 255 (4.18), 204 (4.45) nm; ^1^H and ^13^C NMR data see Table 2; HRESIMS *m*/*z* 355.0469 [M − H]^−^ (calcd for C_18_H_11_O_8_, *m*/*z* 355.0459).

### 3.5. Computational Calculation of ECD Data

The ECD data for selected compounds (**1a** and **1b**) were calculated using Spartan’14 V1.1.4 software (Wavefunction, Inc., Tokyo, Japan) in the MMFF force field with a threshold of 3.0 kJ/mol for conformational searches. The conformers were optimized at the B3LYP/6-31G(d) level in gas phase, and then time-dependent density functional theory (TD-DFT) ECD calculations were performed at the B3LYP/TZVP level for all selected conformers in MeCN. The rotational strengths for 30 excited states were calculated, and ECD spectra were generated using SpecDis version 1.6 (University of Würzburg, Würzburg), applying Gaussian 16 band shapes with a σ of 0.3 eV^2^.

### 3.6. Bioactivity Assays

#### 3.6.1. Neuroprotective Activity Assay

SH-SY5Y cell survival was evaluated according to the reported papers [57]. The cells, obtained from the American Type Culture Collection (ATCC, Manassas, VA, USA) at high passages, maintained at 37 °C in a humidified atmosphere containing 5% CO_2_. Cells were seeded into 96-well plates at a density of 2 × 10^5^ cells/mL in MEM/F12 medium supplemented with 10% (*v*/*v*) fetal bovine serum. Experiments were carried out after 24 h of seeding. Test compounds and positive control (epigallocatechin-3-gallate, EGCG; NAC) were made to 10^−2^ M stock solutions with DMSO and then diluted to corresponding concentrations with cell culture medium. Cells were incubated with test compounds or EGCG (10 μM, Sigma, St. Louis, MO, USA, purity > 98%)/NAC for 2 h, followed by exposure to 80 μM H_2_O_2_ or 10 μM Aβ_25−35_ for another 24 h without medium replacement. After treatment, 10 μL of MTT (5 mg/mL) was added to each well and incubated at 37 °C for 3 h. The cells were finally lysed with 100% DMSO, and the absorbance of MTT formazan was measured at 490 nm using a microplate reader.

#### 3.6.2. Cytotoxicity Assay

The cytotoxic activities of all isolates were evaluated using the CCK-8 assay against five human cancer cell lines: Hela229, SGC7901, PC-3, BEL7402, MCF-7/ADR. The cell lines were obtained from the Cell Bank of the Chinese Academy of Sciences and Shanghai Rongmin Biotechnology Center. All cells were maintained at 37 °C with a 5% CO_2_ humidified atmosphere, using growth medium supplemented with 10% fetal bovine serum, as recommended by the providers. Test compounds were dissolved in DMSO to prepare stock solutions, and subsequently diluted in culture medium for the assays. The final DMSO concentration was less than 0.5% in the assay. Control cells (untreated) were exposed to the same amount of DMSO and incubated under the same conditions. Doxorubicin was used as a positive control.

Cancer cells were seeded in 96-well microculture plates, at a density of 1.5 × 10^4^ cells per well. Each cell line was treated with 100 μM of the test compounds for 48 h. After incubation, 10 μL of CCK-8 solution was added to each well, and the plates were incubated at 37 °C in humidified 5% CO_2_ for 2–4 h. The absorbance at 490 nm was measured with a microplate reader. The inhibition rate of cell proliferation was determined with the following formula: inhibition rate (%) = [(OD_DMSO_ − OD_blank_) − (OD_compound_ − O_blank_)]/(OD_DMSO_ − OD_blank_) ×100%. All data are presented as the mean of three independent experiments. IC_50_ values were calculated by GraphPad Prism 8 software.

#### 3.6.3. Antiviral Assays

Coxsackievirus B. The antiviral activity was tested against several virus strains, including HSV-1 (herpes simplex virus type 1), RSV (respiratory syncytial virus), CVB (coxsackievirus B), EV-71 (enterovirus 71), VSV (vesicular stomatitis virus), HCoV-229E (common human coronavirus), and SARS-CoV-2 (novel coronavirus). Vero cells (African green monkey kidney cells) and Vero-E6 cells were used for infection with virus dilutions. After infection for 2 h at 37 °C, drugs were added, and the cells were incubated for 48–96 h. The cytopathic effect (CPE) was assessed under a microscope, and cell viability was measured using the MTT method. Ribavirin was used as a positive control.

Influenza virus (A/B). Madin–Daby canine kidney (MDCK) cells were purchased from the Cell Bank of Type Culture Collection of Chinese Academy of Science (Shanghai, China). Influenza virus A/H3N2 was provided by Shanghai Municipal Center for Disease Control and Prevention, whereas influenza virus B/Victoria strain was purchased from ATCC. MDCK cells were maintained in DMEM/F12 medium (10%FBS, penicillin/streptomycin, all Gibco, Waltham, MA, USA) at 37 °C in 5% CO_2_ humidified incubator. To form the confluent cell monolayer, 2 × 10^4^ cells/well were seeded into 96-well plates and incubated for 24 h, and 100 μL 2-folder serial dilutions of the analytes in medium were added to each well in triplicate. Culture medium was used as the blank control. The plate was further incubated at 37 °C for 48 h and the maximal nontoxic concentration was selected to evaluate the cytopathic effect (CPE). The CPE assay involved seeding MDCK cells into 96-well plates (2 × 10^4^ cells/well, DMEM/F12, 10% FBS) and incubating them for 24 h in a 5% CO_2_ atmosphere at 37 °C. The culture supernatants were removed before cells were infected with A/H3N2 or B/Victoria (100 × TCID_50_) diluted in infection medium (serum-free DMEM/F12, containing 0.3% trypsin solution) for 2 h at 37 °C. After removing the infection medium, the analyte solutions (diluted in the serum-free DMEM/F12 medium) were added to each well in triplicate. Ribavirin was used as a positive control. CPE was observed (Leica DMI3000B, Wetzlar, Germany) after 72 h incubation and scored at seven levels as follows: 0% CPE, 10% CPE, 25% CPE, 50% CPE, 75% CPE, 90%, and 100%. Cells were washed with sugar-free serum-free DMEM and added to serum-free DMEM/F12 culture medium containing 0.5 mg/mL MTT, incubated for 3.5 h. The supernatant was aspirated and 100 μL of DMSO was added to dissolve the precipitate, and the 490 nm O.D. was measured. The effect of virus inhibition was calculated as (1 − CPE_exp_/CPE_virus control_) × 100%. The cell protective rate was calculated as (OD_exp_ − OD_virus control_)/(OD_normal control_ − OD_virus control_) × 100%.

## 4. Conclusions

Thirty-three (**1**–**33**) structurally diverse secondary metabolites, comprising 15 alkaloids, 4 pulvinic acid derivatives, and 14 ergosterols, were isolated and identified from the fruiting bodies of two edible bolete mushrooms, *P. portentosus* and *B. roseoflavus.* Among the isolates, three (**1**, **2**, and **16**) were new. Particularly, compound **1** possesses a novel framework distinct from *N*-substituted pyrrole alkaloids, featuring a new C-C bond formed between C-6 and C-7′, which highlights the structural diversity of the alkaloids in these mushrooms. The comprehensive investigation into the chemical constituents provides a solid material foundation for the development of boletes as a valuable resource for bioactive compounds. Furthermore, bioactivity assessments revealed significant neuroprotective, cytotoxic effects, and antiviral activities of the isolates. The neuroprotective activities suggest their potential for preventing and treating neurodegenerative diseases, while the cytotoxic and antiviral effects highlight their promising applications in cancer prevention and antiviral therapies.

In summary, the present study not only significantly enriches the structural and bioactivity diversity of secondary metabolites derived from Chinese boletes, but also underscores their potential in addressing neurodegenerative diseases, cancer, and viral infections. This, in turn, paves the way for prospective applications in the development of functional foods and pharmaceuticals. Nevertheless, current research remains limited to preliminary activity screening with no in-depth mechanistic pathway investigations. Further investigations into the underlying mechanisms are warranted, with a particular focus on their anti-influenza virus activity.

## Figures and Tables

**Table 1:** ^1^H (600 MHz) and ^13^C (150 MHz) NMR data of **1**, **2**, and **4**.

No.	1 ^a^		2 ^b^		Inotopyrrole (4) ^b^	
	*δ*_H_ (*J* in Hz)	*δ*_C_	*δ*_H_ (*J* in Hz)	*δ*_C_	*δ*_H_ (*J* in Hz)	*δ*_C_
1	8.52, s					
2		133.5		132.4		132.3
3	6.87, d (3.9)	116.4	6.92, d (4.0)	124.2	6.94, d (4.2)	124.6
4	5.99, d (3.9)	111.3	6.19, d (4.0)	111.3	6.18, d (4.2)	110.1
5		141.8		138.9		144.7
6	3.32, dd (14.6, 7.1)	32.7	4.11, s	65.6	4.30, s	56.3
	3.00, dd (14.6, 7.1)					
1′		130.9		138.6		138.5
2′	6.74, d (2.2)	115.8	7.16, br d (7.7)	129.0	7.11, br d (7.8)	129.1
3′		146.5	7.29, br dd (7.7, 6.9)	128.5	7.27, br t (7.8)	128.6
4′		145.9	7.23, br t (6.9)	126.6	7.22, br t (7.8)	126.7
5′	6.69, d (8.2)	118.1	7.29, br dd (7.7, 6.9)	128.5	7.27, br t (7.8)	128.6
6′	6.60, dd (8.2, 2.2)	120.3	7.16, br d (7.7)	129.0	7.11, br d (7.8)	129.1
7′	3.84, dd (9.2, 7.0)	52.0	3.02, t (7.6)	37.8	3.06, t (7.2)	37.7
8′		175.6	4.51, t (7.6)	47.8	4.56, t (7.2)	47.6
-CHO	9.26, s	179.9	9.58, s	179.4	9.59, s	179.4
-OCH_3_	3.61, s	52.5	3.31, s	57.9		

^a^ Measured in CD_3_OD. ^b^ Measured in CDCl_3_.

**Table 2:** ^1^H (600 MHz) and ^13^C (150 MHz) NMR data of **16** and **18**.

No.	16 ^a^		16 ^b^		Xerocomic Acid (18) ^b^	
	*δ*_H_ (*J* in Hz)	*δ*_C_	*δ*_H_ (*J* in Hz)	*δ*_C_	*δ*_H_ (*J* in Hz)	*δ*_C_
1		126.8		128.8		125.6
2	6.99, br d (8.4)	131.5	7.14, br d (8.4)	132.8	7.26, br d (8.4)	132.6
3	6.71, dd (8.4, 2.0)	113.8	6.78, br d (8.5)	114.7	6.87, br d (8.8)	115.4
4		156.2		157.2		158.3
5	6.71, dd (8.4, 2.0)	113.8	6.78, br d (8.5)	114.7	6.87, br d (8.8)	115.4
6	6.99, br d (8.4)	131.5	7.14, br d (8.4)	132.8	7.26, br d (8.4)	132.6
7		168.9		169.8		167.1
8		95.1		97.2		104.3
9		167.0		169.6		160.6
10		152.0		153.4		155.2
11		117.5		119.2		117.0
12		169.3		170.2		174.0
1′		123.9		126.1		122.7
2′	7.68, br s	113.3	7.89, d (2.0)	114.2	7.76, d (2.0)	115.4
3′		144.5		145.0		145.5
4′		143.0		143.8		146.2
5′	6.67, br d (8.5)	115.1	6.78, br d (8.5)	115.5	6.87, br d (8.8)	116.0
6′	7.46, br d (8.5)	117.0	7.71, dd (8.4, 2.0)	119.2	7.61, dd (8.1, 2.0)	121.0
3′-OH	8.78, dd (6.8, 6.4)					
4′-OH	8.62, dd (12.0, 7.1)					
4-OH	9.35, s					

^a^ Measured in DMSO-*d*_6_. ^b^ Measured in acetone-*d*_6_.

## Data Availability

Data and figures generated or used in this study appear in the submitted article.

## Supplementary Materials

The following supporting information can be downloaded at https://www.mdpi.com/article/10.3390/molecules30061197/s1, Tables S1–S5: bioactivity assays data. Figures S1−S3: compounds separation flowchart, Figures S4–S21: original 1D-/2D NMR, HRESIMS spectra for the new compounds, and spectroscopic data for known compounds.
