# Supplementary material for: Bioactive Secondary Metabolites of Two Chinese Edible Boletes, Phlebopus portentosus and Butyriboletus roseoflavus
Source: Molecules. 2025 Mar 7;30(6):1197. doi: 10.3390/molecules30061197 (PMC11944884; doi:10.3390/molecules30061197)
Supplement: Supplementary file 1 [file molecules-30-01197-s001.zip › molecules-3484709-supplementary.pdf]

Supplementary Data for

Bioactive Secondary Metabolites of Two Chinese Edible Boletes, *Phlebopus portentosus* and *Butyriboletus roseoflavus*

Zhixuan Wang [1], Wei Zhou [2], Yuhang He [1], Zeyu Zhao [1], Yang Cao [3,4], Shunzhen Luo [3], Guangyan Ji [3], Kaiping Ji [3,4], Jing Chen [5,*], Jiyang Li [1], Juan Xiong [1,*]

[1] School of Pharmacy, Fudan University, Shanghai 201203, China

[2] Department of Chemistry, Fudan University, Shanghai 200438, China

[3] Jinghong Hongzhen Agricultural Science and Technology Co. Ltd., Jinghong, Yunnan 666100, China

[4] Guizhou Hongzhen Fungus Industry Investment and Development Co. Ltd, Zhenfeng, Guizhou 562200, China

[5] State Key Laboratory of Drug Research, Shanghai Institute of Materia Medica, Chinese Academy of Sciences, Shanghai 201203, China

[*] Corresponding authors.

E-mail addresses: j20-chenjing-nj@simm.ac.cn (JC); jxiong@fudan.edu.cn (JX).

Contents

Table S1. Neuroprotective effect of **18** against Aβ_{25-35}-induced damage in SH-SY5Y cells....2

Table S2. Neuroprotective effect of **19** against H_2O_2-induced damage in SH-SY5Y cells. 2

Table S3. Cytotoxicity of tested compounds on different cancer cells..................................3

Table S4. IC_{50} values of **9** and **33** against carcinoma cells. 3

Table S5. In vitro antiviral effects of **22** and **26** against influenza virus. 4

Fig. S1. Compounds separation flowchart of artificially *P. portentosus*. 5

Fig. S2. Compounds separation flowchart of wild *P. portentosus*..6

Fig. S3. Compounds separation flowchart of wild *B. roseoflavus*..7

Fig. S4. ^{1}H NMR spectrum of compound **1** in CD_3OD (600 MHz)..8

Fig. S5. ^{13}C NMR spectrum of compound **1** in CD_3OD (150 MHz).......................................8

Fig. S6. HMBC spectrum of compound **1** in CD_3OD (600 MHz)..9

Fig. S7. HRESIMS report of compound **1** 9

Fig. S8. ^{1}H NMR spectrum of compound **2** in $CDCl_3$ (400 MHz)..10

Fig. S9. ^{13}C NMR spectrum of compound **2** in $CDCl_3$ (150 MHz). 10

Fig. S10. HRESIMS report of compound **2**...11

Fig. S11. ^{1}H NMR spectrum of compound **16** in DMSO-d_6 (600 MHz)...............................11

Fig. S12. ^{1}H NMR spectrum of compound **16** in acetone-d_6 (600 MHz) 12

Fig. S13. ^{13}C NMR spectrum of compound **16** in DMSO-d_6 (150 MHz)..............................12

Fig. S14. ^{13}C NMR spectrum of compound **16** in acetone-d_6 (150 MHz) 13

Fig. S15. HMBC spectrum of compound **16** in DMSO-d_6 (600 MHz).................................13

Fig. S16. ROESY spectrum of compound **16** in DMSO-d_6 (600 MHz) 14

Fig. S17. HRESIMS report of compound **16**...14

Fig. S18. ^{1}H NMR spectrum of compound **8** in CD_3OD (600 MHz)....................................15

Fig. S19. ^{13}C NMR spectrum of compound **8** in CD_3OD (150 MHz)...................................15

Fig. S20. HMBC spectrum of compound **8** in CD_3OD (600 MHz).......................................16

Fig. S21. HRESIMS report of compound **8**...16

Spectroscopic data of known compounds...17

Table S1. Neuroprotective effect of **18** against A$\beta_{25\text{-}35}$-induced damage in SH-SY5Y cells.

Groups	Cell viability (% of control group) Mean ± SEM				
Control	100.00 ± 0.00				
Model-A$\beta_{25\text{-}35}$ (10 μM)	77.08 ± 1.86$^{\#\#\#}$				
Positive control-EGCG (10 μM)	104.80 ± 2.81***				
Compd.	Final concentration (μM)				
	2.5	5.0	10.0	20.0	40.0
**18**	78.15 ± 1.53	77.60 ± 1.97	80.35 ± 1.39	81.15 ± 1.79	90.50 ± 2.63***

$^{\#\#\#}$p < 0.001 vs control. ***p < 0.001 vs A$\beta_{25\text{-}35}$ groups

Table S2. Neuroprotective effect of **19** against H_2O_2-induced damage in SH-SY5Y cells.

Groups	Cell viability (% of control group) Mean ± SEM				
Control	100.00 ± 0.00				
Model-H_2O_2 (80 μM)	55.85 ± 1.67$^{\#\#\#}$				
Positive control-NAC (500 μM)	81.05 ± 2.28***				
Compd.	Final concentration (μM)				
	2.5	5.0	10.0	20.0	40.0
**19**	56.43 ± 1.79	59.28 ± 2.94	63.70 ± 2.80	71.50 ± 1.78***	81.93 ± 2.93***

$^{\#\#\#}p < 0.001$ vs control. ***$p < 0.001$ vs H_2O_2 groups

Table S3. Cytotoxicity of tested compounds on different cancer cells.

Compd.	Concentration (μM)	Cell proliferation inhibition (%)				
		Hela229	SGC7901	PC-3	BEL7402	MCF-7/ADR
**9**	6.25	37.1	13.2	3.0	12.5	-0.9
	12.5	53.7	17.5	1.7	24.4	1.7
	25	68.7	27.1	4.0	44.4	3.4
	50	83.3	37.4	2.6	56.4	2.9
	100	88.0	44.3	14.0	63.6	2.5
**33**	6.25	-4.8	8.3	12.4	10.0	5.5
	12.5	5.8	16.7	21.6	15.3	5.1
	25	52.3	39.1	47.3	49.4	27.1
	50	96.2	93.5	67.7	75.3	42.6
	100	96.7	94.7	85.9	82.8	81.6
DOX[a]	Con. A/Con. B					
	0.3125/6.25	33.5[b]	26.3[b]	15.2[b]	15.4[b]	46.5[c]
	0.625/12.5	41.5[b]	33.1[b]	40.0[b]	19.6[b]	44.2[c]
	1.25/25	57.6[b]	71.5[b]	41.1[b]	38.8[b]	48..6[c]
	2.5/50	82.9[b]	82.0[b]	63.2[b]	69.2[b]	52.7[c]
	5/100	93.1[b]	92.6[b]	60.7[b]	80.1[b]	52.8[c]

[a] Doxorubicin (DOX) was posive control.

[b] The test concentrations were Con.A.

[c] The test concentrations were Con.B.

Table S4. IC_{50} values of **9** and **33** against carcinoma cells.

Compd.	IC_{50} (μM)				
	Hela229	SGC7901	PC-3	BEL7402	MCF-7/ADR
**9**	10.76	n.a.[b]	n.a.[b]	41.54	n.a.[b]
**33**	24.45	26.66	28.43	27.72	n.a.[b]
DOX[a]	0.75	0.80	1.78	1.59	35.2

[a] Doxorubicin (DOX) was posive control.

[b] No activity (n.a., IC_{50} > 50 μM).

Table S5. In vitro antiviral effects of **22** and **26** against influenza virus.

Compd.	Concentration (μM)	A/H3N2 strain		B/Victoria strain	
		CPE inhibition	IC_{50}	CPE inhibition	IC_{50}
**22**	1.56	0%	9.6 μM	0%	6.6 μM
	3.13	0%		0%	
	6.25	0%		10%	
	12.5	75%		75%	
	25	75%		75%	
**26**	0.78	3.3%	4.0 μM	6.6%	3.6 μM
	1.56	15%		15%	
	3.13	27.5%		25%	
	6.25	75%		75%	
	12.5	82.5%		75%	
**Ribavirin**[a]	3.13	3.3%	28.3 μM	0%	20.6 μM
	6.25	0%		0%	
	12.5	10%		25%	
	25	50.5%		62.5%	
	50	75%		75%	
	100	100%		100%	

[a] positive control.

Fig. S1. Compounds separation flowchart of artificially *P. portentosus*.

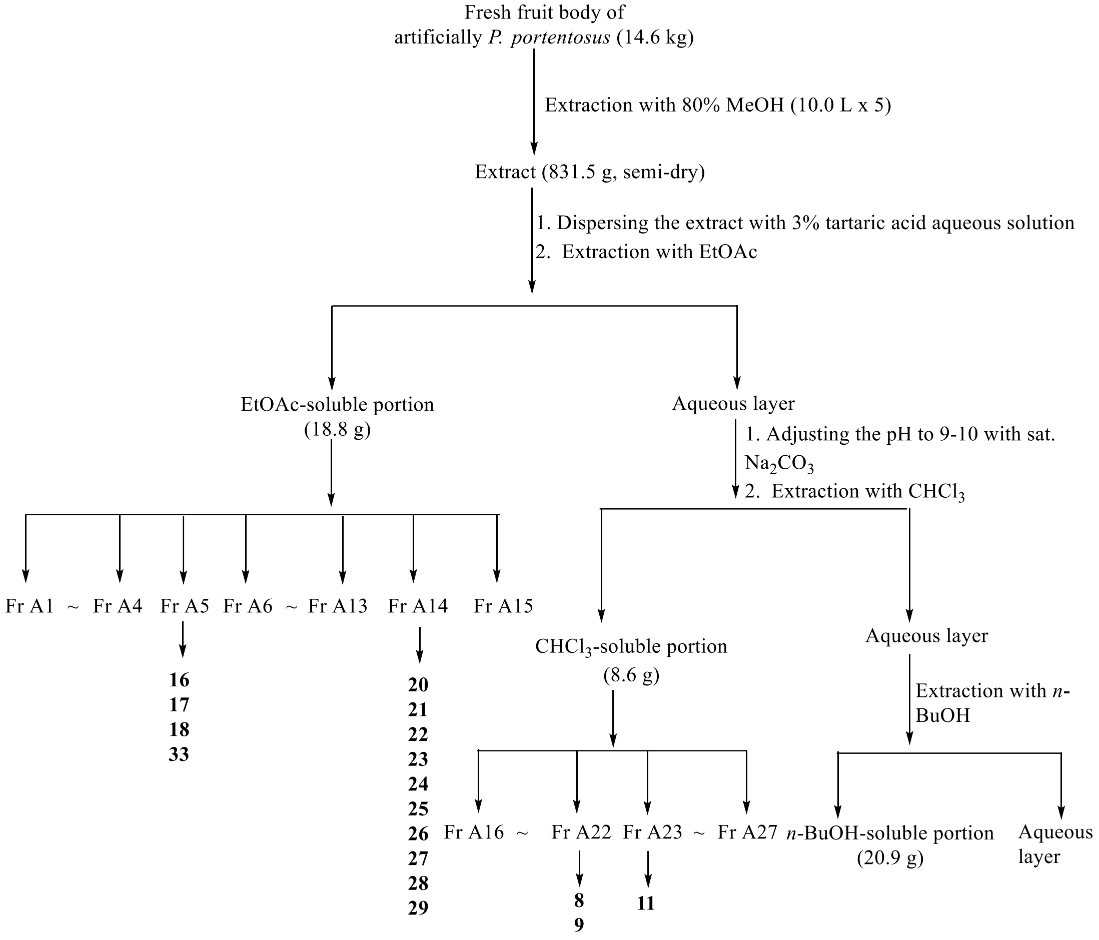

Fig. S2. Compounds separation flowchart of wild *P. portentosus*.

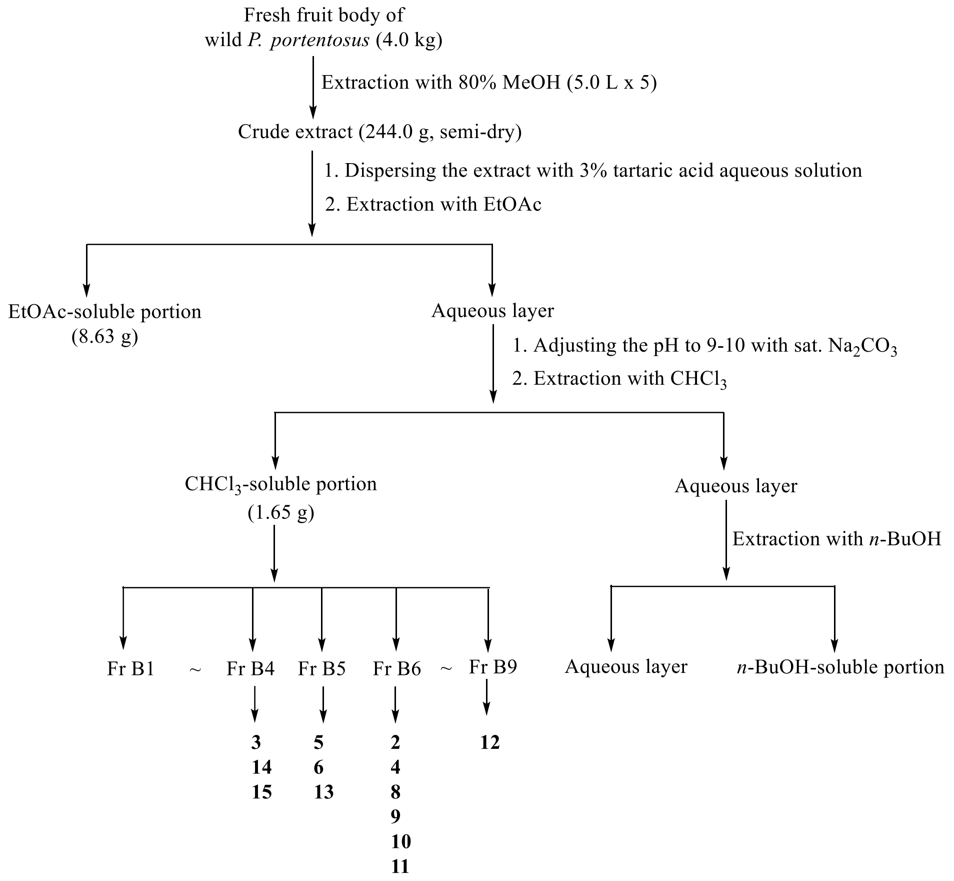

Fig. S3. Compounds separation flowchart of *B. roseoflavus*.

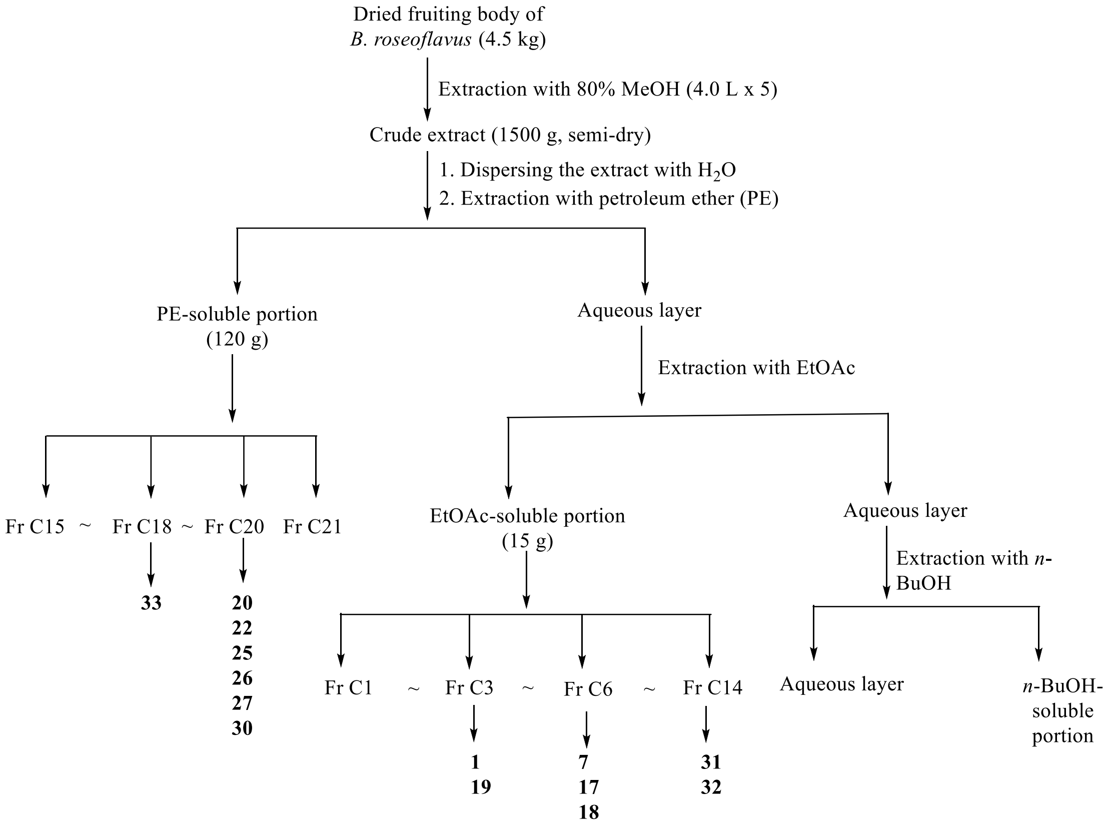

Fig. S4. ^{1}H NMR spectrum of compound **1** in CD_3OD (600 MHz).

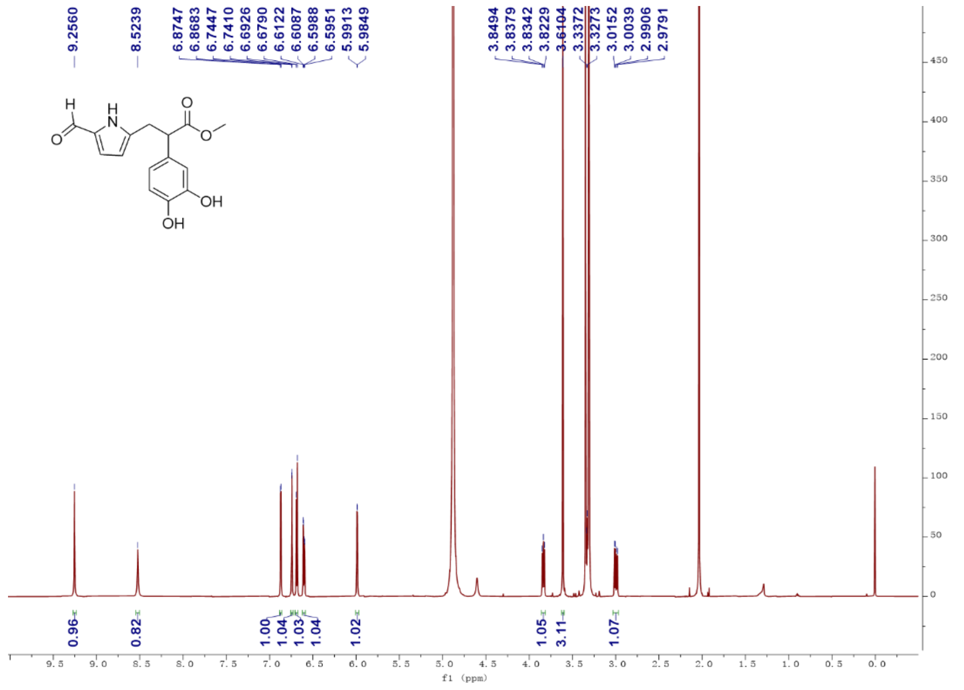

Fig. S5. ^{13}C NMR spectrum of compound **1** in CD_3OD (150 MHz).

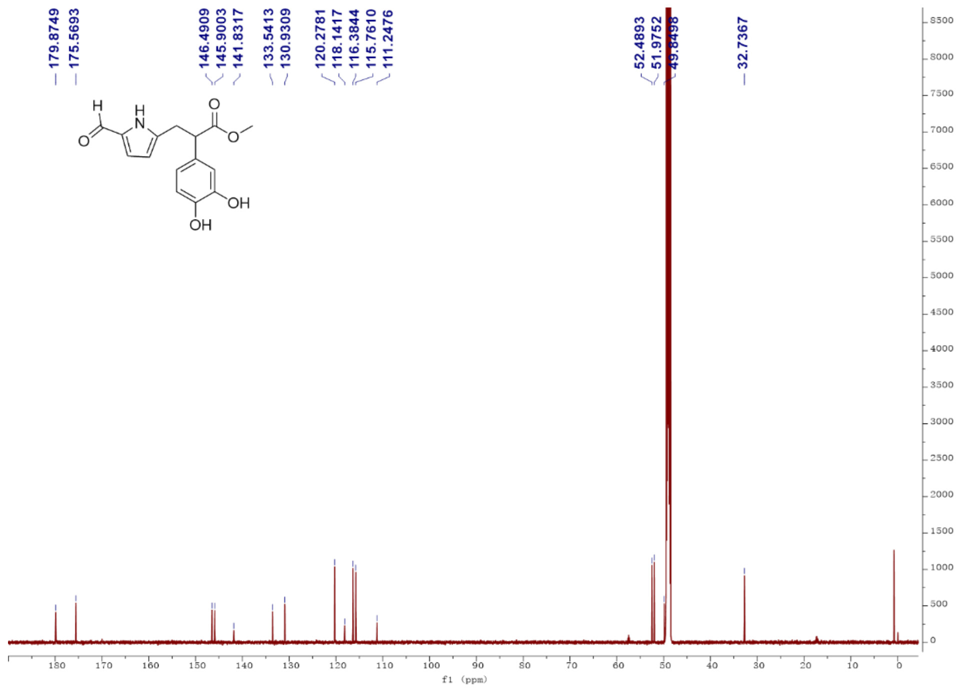

Fig. S6. HMBC spectrum of compound **1** in CD_3OD (600 MHz)

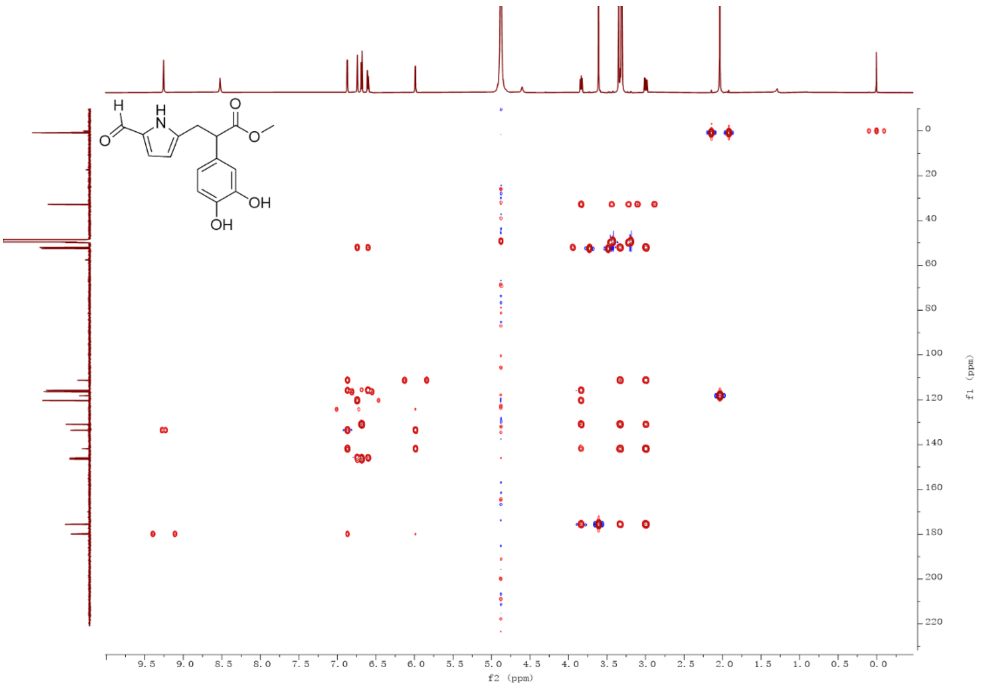

Fig. S7. HRESIMS report of compound **1**

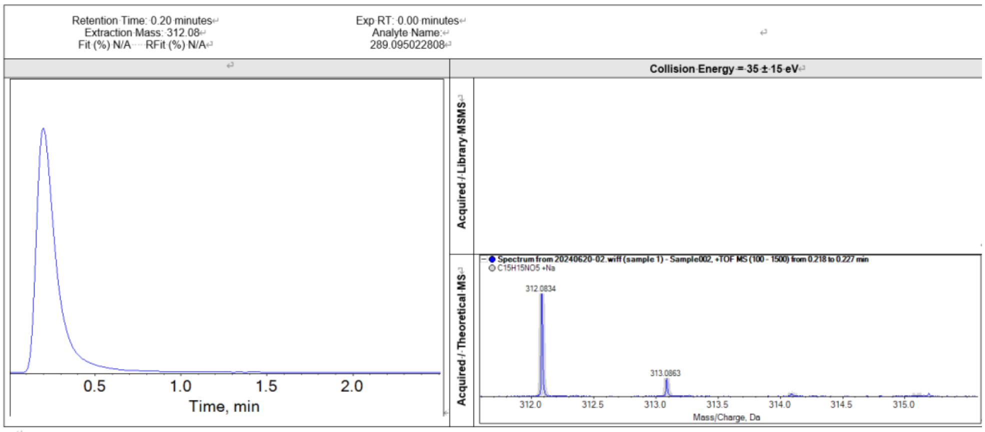

	Compound Name (Library Hit)	Score	Formula	Intensity	Threshold	Expected m/z	Found at m/z	Error (ppm)	Expected RT (min)	Found RT (min)	RT Delta (min)	Isotope Diff (%)	Library Score (%)
✓ ● ✓ ● ●	289.095022808 (No Acquired MSMS)	89%	C15H15NO5	834243	5	312.0842	312.0834	-2.6	0.00	0.20	0.20	0.5%	N/A

Fig. S8. ^{1}H NMR spectrum of compound **2** in $CDCl_3$ (400 MHz).

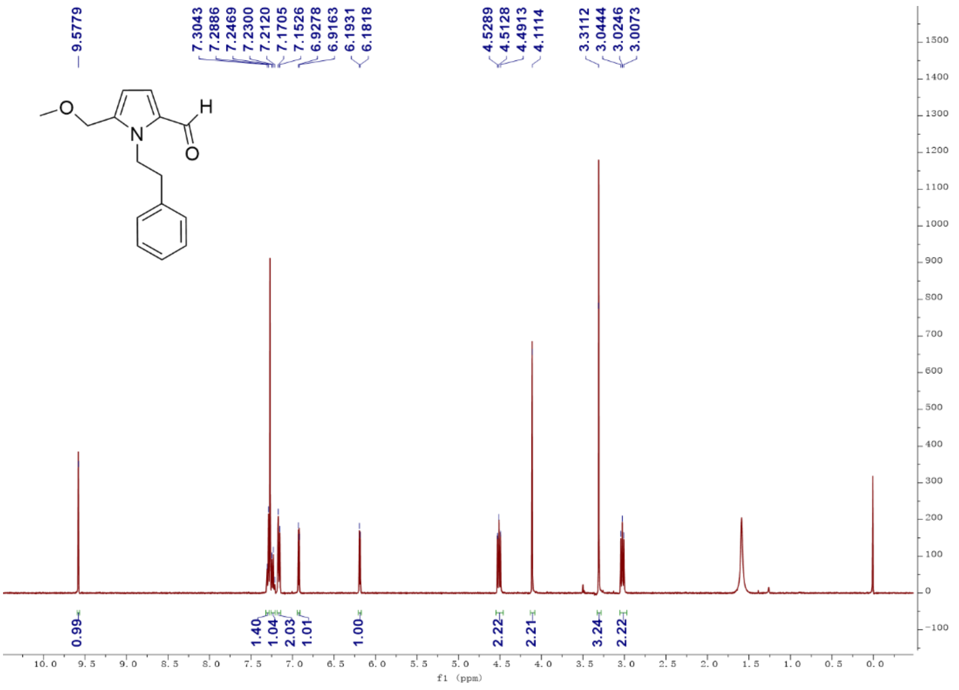

Fig. S9. ^{13}C NMR spectrum of compound **2** in $CDCl_3$ (150 MHz).

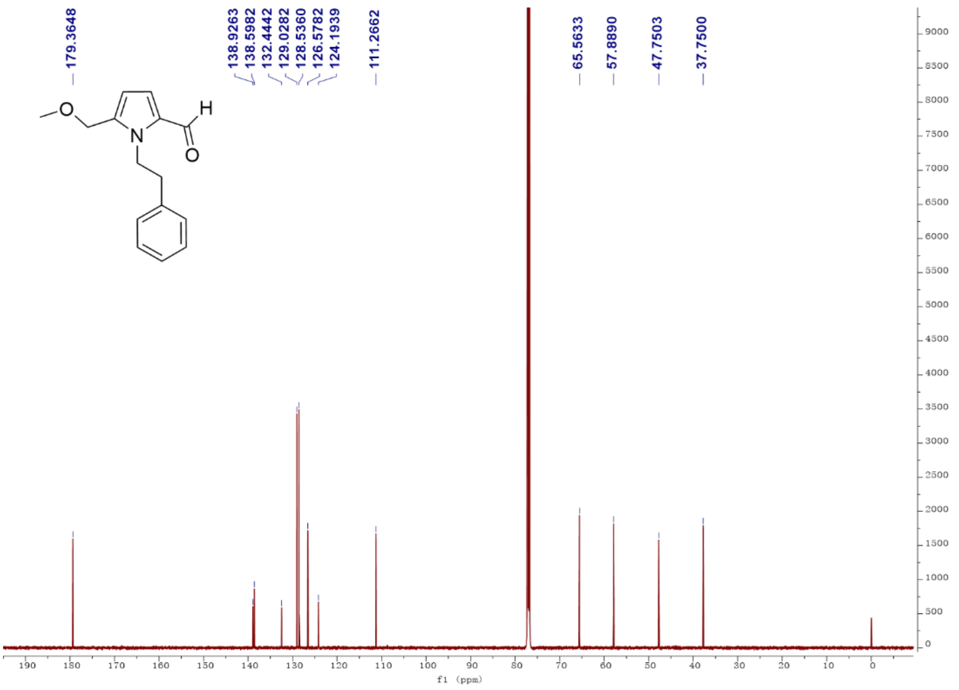

Fig. S10. HRESIMS report of compound **2**

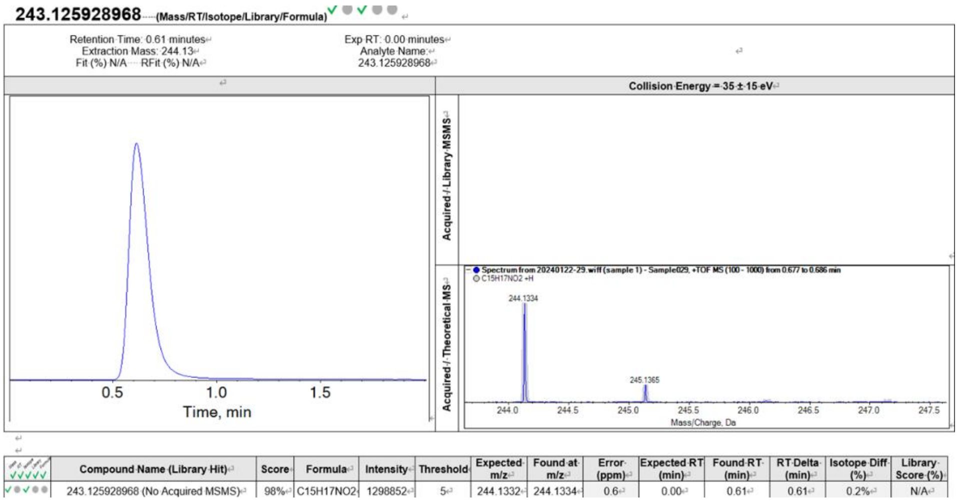

Fig. S11. ^{1}H NMR spectrum of compound **16** in DMSO-d_6 (600 MHz)

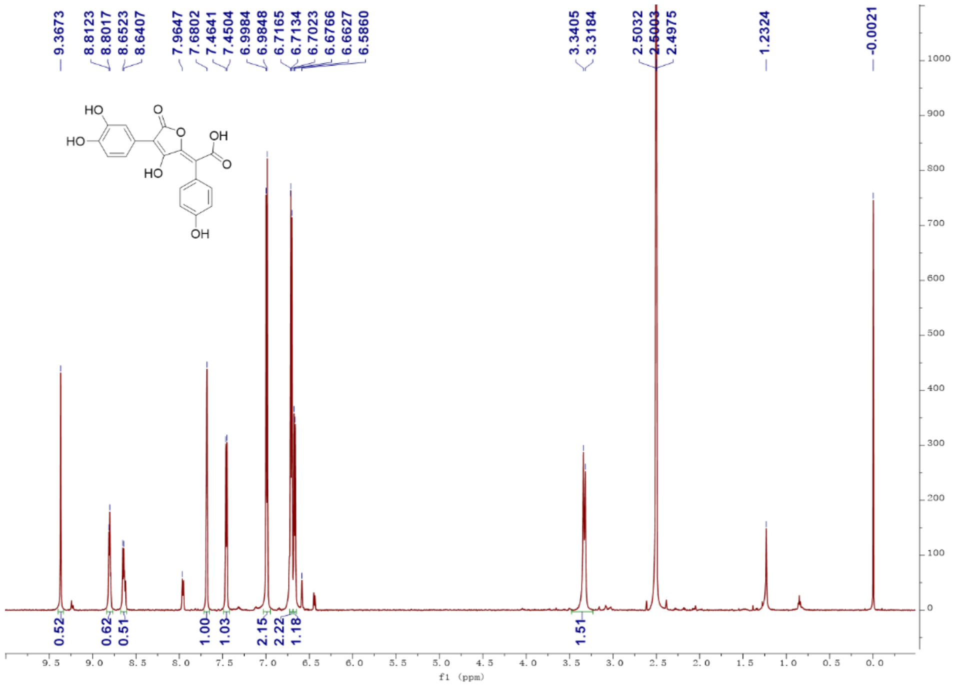

Fig. S12. ^{1}H NMR spectrum of compound **16** in acetone-d_6 (600 MHz)

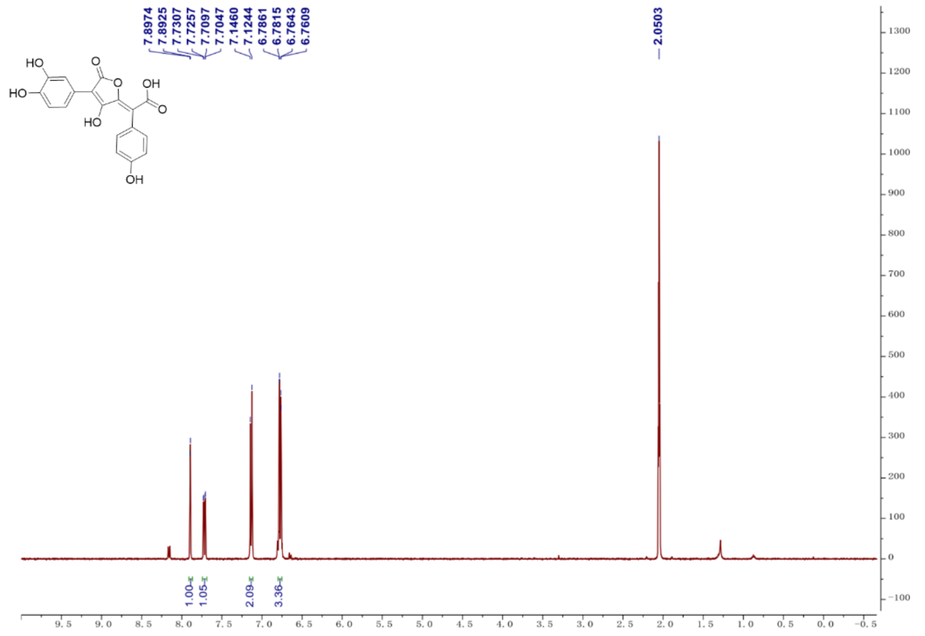

Fig. S13. ^{13}C NMR spectrum of compound **16** in DMSO-d_6 (150 MHz)

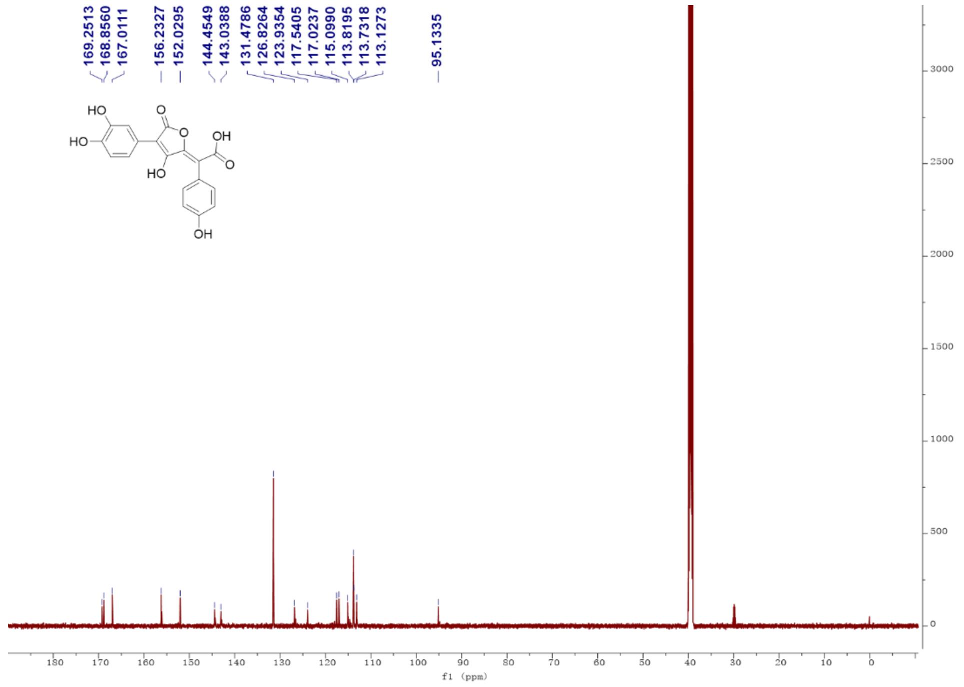

Fig. S14. ^{13}C NMR spectrum of compound **16** in acetone-d_6 (150 MHz)

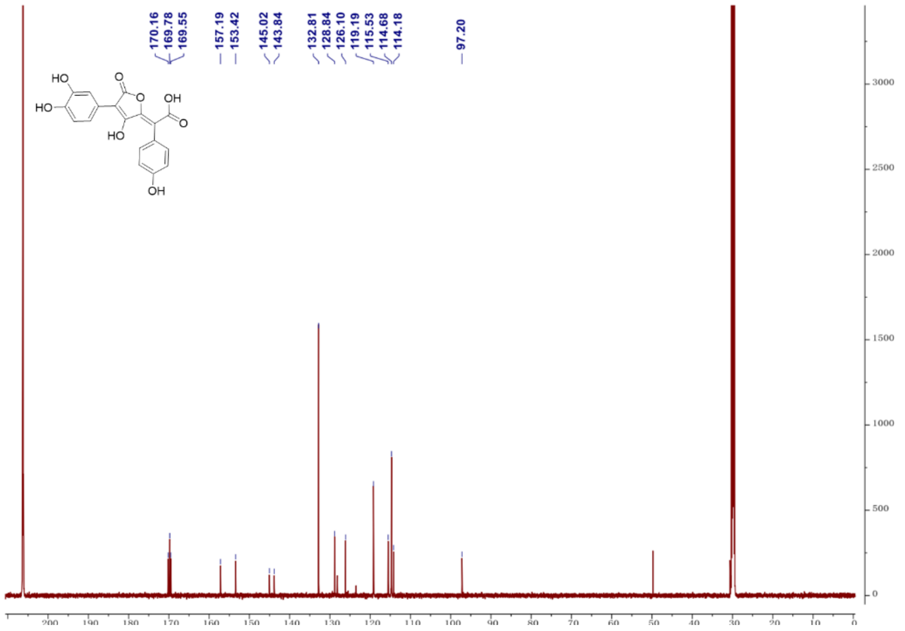

Fig. S15. HMBC spectrum of compound **16** in DMSO-d_6 (600 MHz)

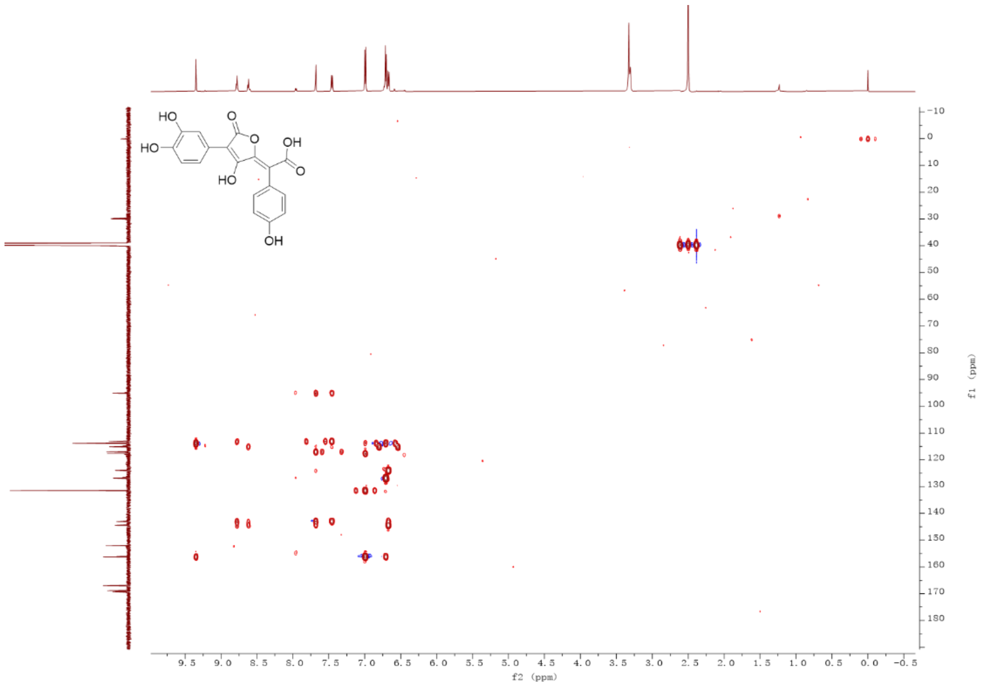

Fig. S16. ROESY spectrum of compound **16** in DMSO-d_6 (600 MHz)

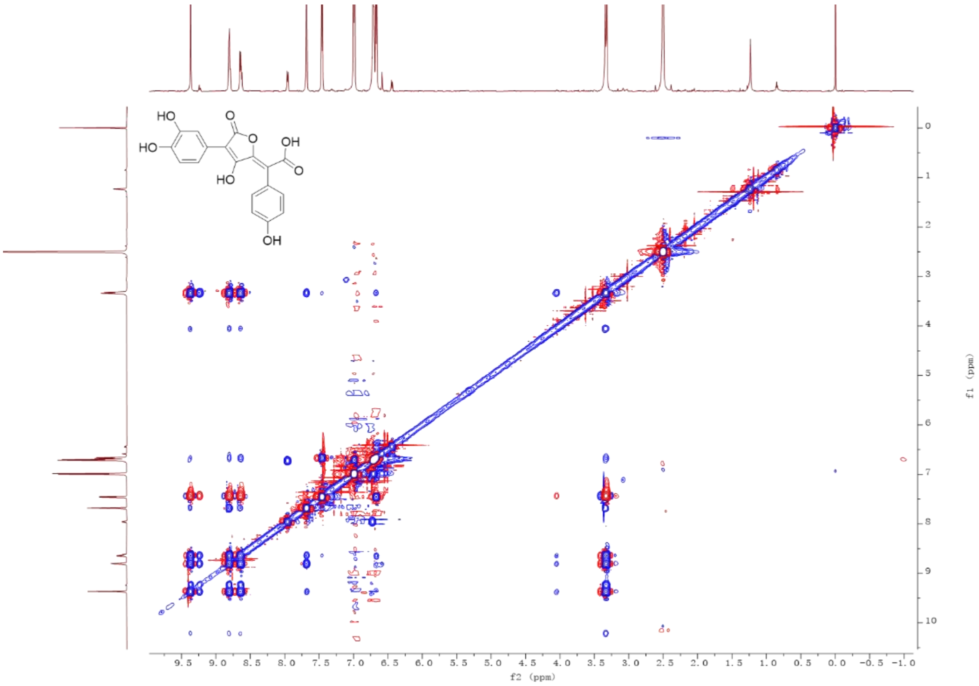

Fig. S17. HRESIMS report of compound **16**

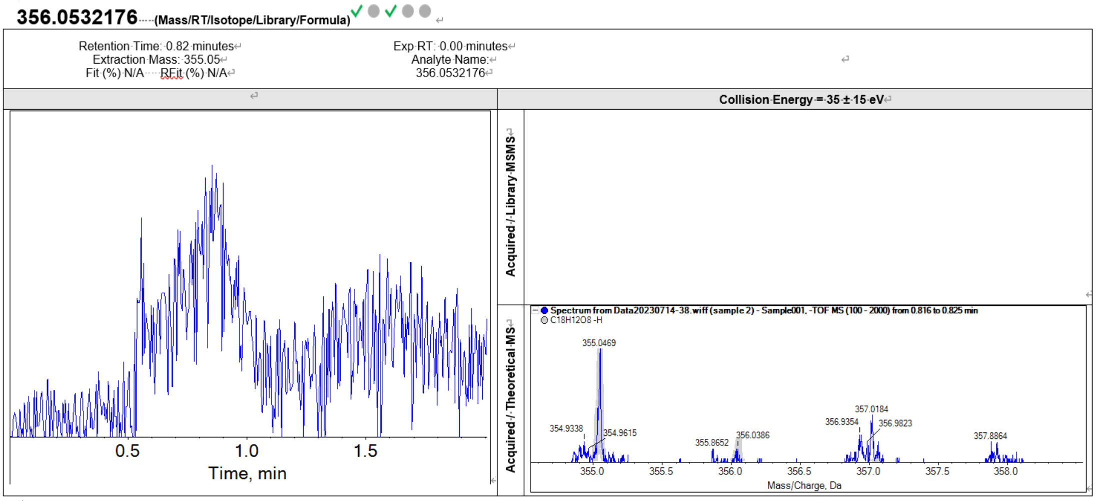

	Compound Name (Library Hit)	Score	Formula	Intensity	Threshold	Expected m/z	Found at m/z	Error (ppm)	Expected RT (min)	Found RT (min)	RT Delta (min)	Isotope Diff (%)	Library Score (%)
	356.0532176 (No Acquired MSMS)	83%	C18H12O8	309	5	355.0459	355.0469	2.7	0.00	0.82	0.82	20.5%	N/A

Fig. S18. ^{1}H NMR spectrum of compound **8** in CD_3OD (600 MHz).

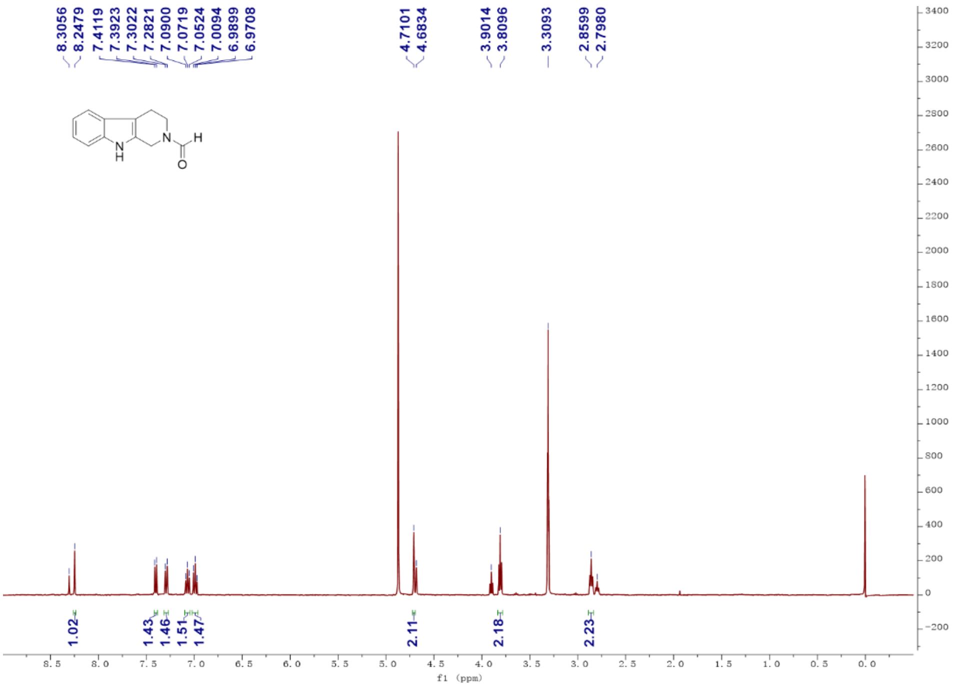

Fig. S19. ^{13}C NMR spectrum of compound **8** in CD_3OD (150 MHz).

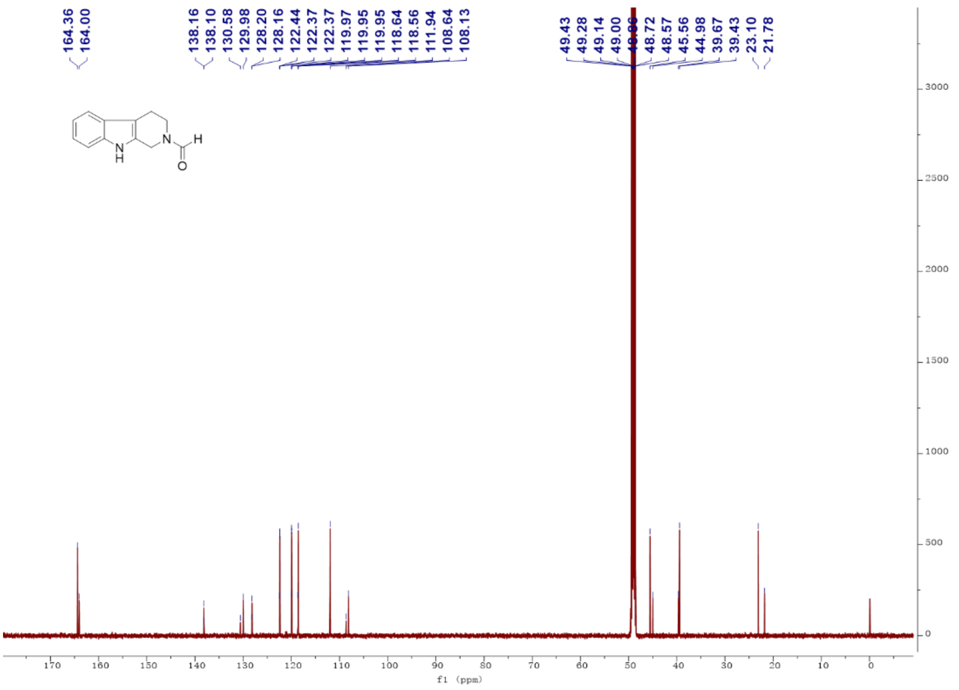

Fig. S20. HMBC spectrum of compound **8** in CD_3OD (600 MHz)

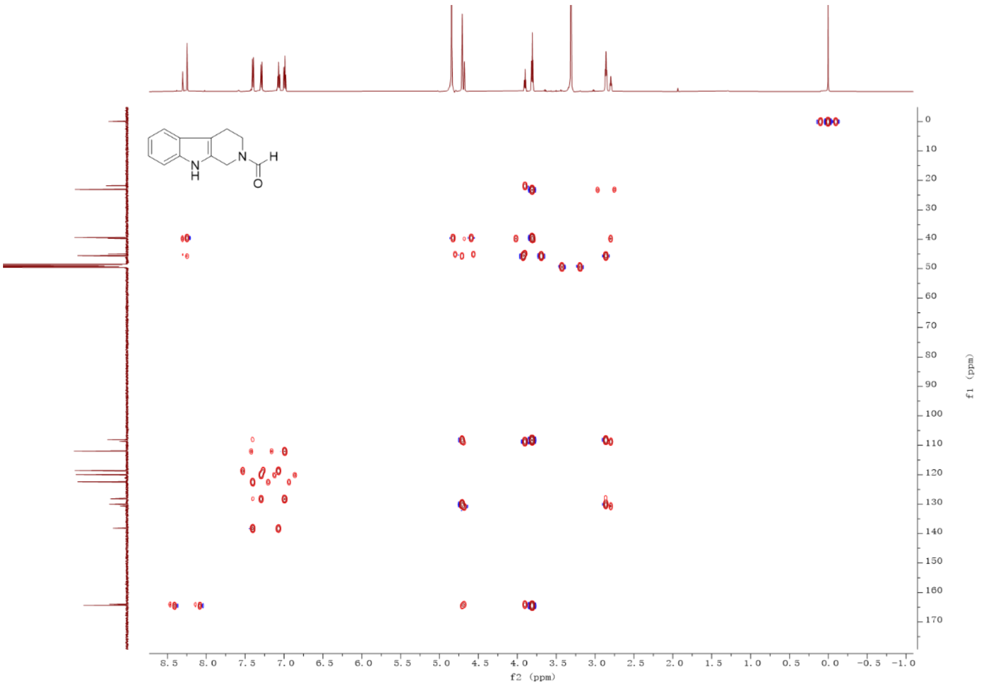

Fig. S21. HRESIMS report of compound **8**

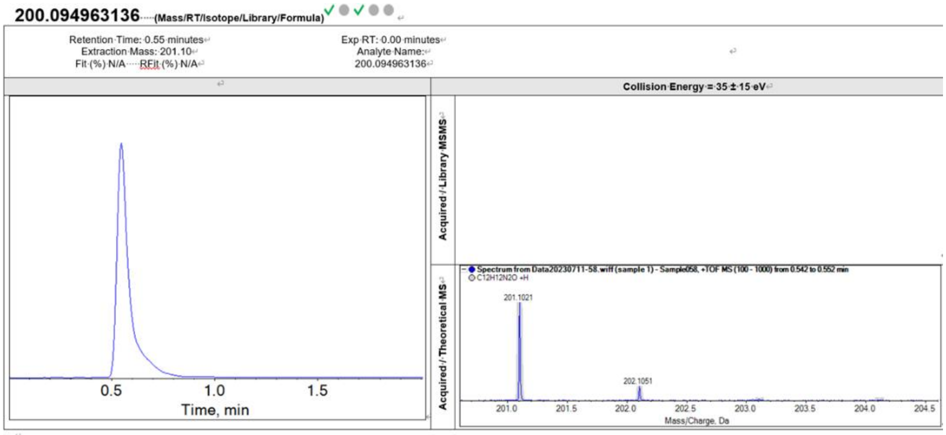

	Compound Name (Library Hit)	Score	Formula	Intensity	Threshold	Expected m/z	Found at m/z	Error (ppm)	Expected RT (min)	Found RT (min)	RT Delta (min)	Isotope Diff (%)	Library Score (%)
✓ ● ✓ ● ●	200.094963136 (No Acquired MSMS)	98%	C12H12N2O	836973	5	201.1022	201.1021	-0.7	0.00	0.55	0.55	0.3%	N/A

Spectroscopic data of known compounds

Pyrrolezanthine (3)

Amorphous powder; ^{1}H NMR ($CDCl_3$, 400 MHz): δ_H 9.57 (1H, s, -CHO), 6.95 (2H, d, *J* = 8.0 Hz, H-2′ & 6′), 6.93 (1H, d, *J* = 4.0 Hz, H-3), 6.74 (2H, d, *J* = 8.0 Hz, H-3′ & H-5′), 6.18 (1H, d, *J* = 4.0 Hz, H-4), 4.50 (2H, t, *J* = 7.3 Hz, H_2-8′), 4.31 (2H, s, H_2-6), 2.97 (2H, t, *J* = 7.3 Hz, H_2-7′). (+)-ESI-MS: *m/z* 246 $[M + H]^+$.

Inotopyrrole (4)

Amorphous powder; ^{1}H NMR ($CDCl_3$, 400 MHz): δ_H 9.59 (1H, s,-CHO), 7.27 (2H, t, *J* = 8.4 Hz, H-3′ & H-5′), 7.22 (1H, t, *J* = 8.4 Hz, H-4′), 7.11 (2H, d, *J* = 7.8 Hz, H-2′ & H-6′), 6.94 (1H, d, *J* = 4.0 Hz, H-3), 6.18 (1H, d, *J* = 4.0 Hz, H-4), 4.50 (2H, t, *J* = 7.2 Hz, H_2-8′), 4.30 (2H, s, H_2-6), 3.06 (2H, t, *J* = 7.2 Hz, H_2-7′). ^{13}C NMR ($CDCl_3$, 150 MHz): δ_C 179.4 (-CHO), 144.7 (C-5), 138.5 (C-1′), 132.3 (C-2), 129.1 (C-2′ & C-6′), 128.6 (C-3′ & C-5′), 126.7 (C-4′), 124.6 (C-3), 110.1 (C-4), 56.3 (C-6), 47.6 (C-8′), 37.7 (C-7′). (+)-ESI-MS: *m/z* 230 $[M + H]^+$.

Phlebopine B (5)

Pale yellow amorphous powder; $[\alpha]_D^{20}$ +10.3 (*c* 0.10, $CHCl_3$), ^{1}H NMR (DMSO-d_6, 400 MHz): δ_H 9.43 (1H, s, -CHO), 6.97 (1H, br s, H-3), 6.22 (1H, br s, H-4), 4.49 (2H, s, H_2-6), 4.10 (1H, m, H-1′a), 4.19 (1H, m, H-1′b), 1.78 (1H, m, H-2′), 1.23 (1H, m, H-3′a), 1.13 (1H, m, H-3′b), 0.84 (3H, t, *J* = 7.4 Hz, H_3-4′), 0.73 (3H, t, *J* = 6.6 Hz, H_3-5′); (+)-ESI-MS: *m/z* 196 $[M + H]^+$.

Ganoine (6)

Pale yellow amorphous powder; ^{1}H NMR ($CDCl_3$, 400 MHz): δ_H 9.53 (1H, s, -CHO), 6.87 (1H, br s, H-3), 6.23 (1H, br s, H-4), 4.69 (2H, s, H_2-6), 4.38 (2H, t, *J* = 7.5 Hz, H_2-1′), 1.71 (2H, m, H_2-2′), 1.64 (1H, m, H-3′), 0.98 (6H, d, *J* = 6.6 Hz, H_3-4′ & H_3-5′); (+)-ESI-MS: *m/z* 196 $[M + H]^+$.

4-(2-Formyl-5-(methoxymethyl)-1*H*-pyrrol-1-yl) butanoic acid (7)

Pale yellow amorphous powder; ^{1}H NMR ($CDCl_3$, 400 MHz): δ_H 9.48 (1H, s, -CHO), 6.89 (1H, d, *J* = 4.0 Hz, H-3), 6.24 (1H, d, *J* = 4.0 Hz, H-4), 4.46 (2H, s, H_2-6), 4.39 (2H, t, *J* = 7.6 Hz, H_2-1′), 3.35 (3H, s, 6-OCH_3), 2.41 (2H, t, *J* = 7.1 Hz, H_2-3′), 2.05 (2H, m, H_2-2′); (+)-ESI-MS: *m/z* 248 $[M + Na]^+$.

1,2,3,4-Tetrahydro-1-oxo-*β*-carboline (9)

Pale yellow amorphous powder; ^{1}H NMR ($CDCl_3$, 400 MHz): δ_H 7.61 (1H, d, *J* = 8.2 Hz, H-5), 7.47 (1H, d, *J* = 8.1 Hz, H-8), 7.33 (1H, ddd, *J* = 7.8, 7.6, 1.0 Hz, H-7), 7.17 (1H, ddd, *J* = 7.7, 7.3, 1.0 Hz, H-6), 5.91 (1H, br s, -NH), 3.73 (2H, td, *J* = 7.0, 2.5 Hz, H_2-3), 3.08 (2H, t, *J* = 7.0 Hz, H_2-4). (+)-ESI-MS: *m/z* 187 $[M + H]^+$.

1,2,3,4-Tetrahydro-*β*-carboline (10)

Pale yellow amorphous powder; ^{1}H NMR ($CDCl_3$, 400 MHz): δ_H 7.79 (1H, s, H-9), 7.49 (1H, d, J = 7.5 Hz, H-5), 7.32 (1H, d, J = 7.9 Hz, H-8), 7.10 (1H, dd, J = 7.5, 7.3 Hz, H-6), 7.10 (1H, dd, J = 7.9, 7.3 Hz, H-7), 4.05 (2H, s, H_2-1), 3.20 (2H, t, J = 5.9 Hz, H_2-3), 2.77 (2H, t, J = 5.9 Hz, H_2-4). (+)-ESI-MS: *m/z* 173 $[M + H]^+$.

***β*-Carboline (11)**

Brown amorphous powder; ^{1}H NMR ($CDCl_3$, 600 MHz): δ_H 8.93 (1H, s, H-1), 8.59 (1H, s, 9-NH), 8.47 (1H, d, J = 5.0 Hz, H-3), 8.15 (1H, d, J = 8.1 Hz, H-5), 7.98 (1H, d, J = 5.2 Hz, H-4), 7.57 (1H, t, J = 7.2 Hz, H-7), 7.54 (1H, d, J = 7.2 Hz, H-8), 7.32 (1H, t, J = 7.2 Hz, H-6); ^{13}C NMR ($CDCl_3$, 150 MHz) δ_C 140.4 (C-8a), 139.2 (C-3), 135.9 (C-1), 133.7 (C-9a), 129.0 (C-4a), 128.5 (C-5a), 121.8 (C-7), 121.5 (C-5), 120.2 (C-6), 114.7 (C-4), 111.5 (C-8); (+)-ESI-MS: *m/z* 169 $[M + H]^+$.

Butyl 1*H*-imidazole-4-carboxylate (12)

Pale yellow amorphous powder; ^{1}H NMR (DMSO-d_6, 400 MHz): δ_H 7.72 (1H, m, H-5), 7.66 (1H, m, H-2), 4.22 (2H, t, J = 6.6 Hz, H_2-7), 1.64 (2H, m, H_2-8), 1.37 (2H, m, H_2-9), 0.91 (3H, t, J = 7.3 Hz, H_3-10); (+)-ESI-MS: *m/z* 169 $[M + H]^+$, *m/z* 191 $[M + Na]^+$.

2-Ethylhexyl 1*H*-imidazole-4-carboxylate (13)

Pale yellow amorphous powder; $[\alpha]_D^{25}$ +8.5 (c 0.08, $CHCl_3$); ^{1}H NMR ($CDCl_3$, 400 MHz): δ_H 7.71 (1H, br s, H-5), 7.54 (1H, br s, H-2), 4.22 (2H, m, H_2-7), 1.69 (1H, m, H-8), 1.42 (2H, m, H_2-1′), 1.38-1.25 (6H, m, H_2-9, H_2-2′, H_2-3′), 0.93 (3H, t, J = 7.8 Hz, H_3-4′), 0.91 (3H, t, J = 7.8 Hz, H_3-10); ^{13}C NMR ($CDCl_3$, 150 MHz): δ_C 167.8 (C-6), 132.4 (C-4), 130.9 (C-2), 128.8 (C-5), 68.2 (C-7), 38.7 (C-8), 30.4 (C-9), 28.9 (C-3′), 23.7 (C-1′), 23.0 (C-2′), 13.9 (C-4′), 10.9 (C-10); (+)-ESI-MS: *m/z* 225 $[M + H]^+$.

***N*-Phenethylformamide (14)**

Pale yellow amorphous powder; exist as a pair of inseparable isomer (**14a**:**14b** = 5:1); ^{1}H NMR ($CDCl_3$, 400 MHz) of **14a**: δ_H 8.15 (1H, br s, H-10), 7.38-7.17 (5H, m, H-2~H-6), 3.60 (2H, q, J = 6.6 Hz, H_2-8), 2.86 (2H, t, J = 6.9 Hz, H_2-7); ^{1}H NMR ($CDCl_3$, 400 MHz) of **14b**: δ_H 7.96 (1H, d, J = 11.7 Hz, H-10), 7.38-7.17 (5H, m, H-2~H-6), 3.50 (2H, q, J = 6.7 Hz, H_2-8), 2.83 (2H, t, J = 6.7 Hz, H_2-7); (+)-ESI-MS: *m/z* 150 $[M + H]^+$.

***N*-Phenethylacetamide (15)**

Pale yellow amorphous powder; ^{1}H NMR ($CDCl_3$, 400 MHz): δ_H 7.35-7.26 (2H, m, H-2 & H-6), 7.25-7.16 (3H, m, H-3~H-5), 5.87 (1H, s, -NH), 3.52 (2H, q, J = 6.6 Hz, H_2-8), 2.83 (2H, t, J = 7.0 Hz, H_2-7), 1.95 (3H, s, H_3-11); (+)-ESI-MS: *m/z* 164 $[M+H]^+$, *m/z* 186 $[M + Na]^+$.

Atromentic acid (17)

Yellow amorphous powder; ^{1}H NMR (acetone-d_6, 400 MHz): δ_H 8.15 (2H, d, *J* = 8.5 Hz, H-2' & H-6'), 7.17 (2H, d, *J* = 8.5 Hz, H-2 & H-6), 6.81 (2H, d, *J* = 8.0 Hz, H-3 & H-5), 6.79 (2H, d, *J* = 8.0 Hz, H-3' & H-5'); (−)-ESI-MS: *m/z* 339 $[M - H]^-$.

Xerocomic acid (18)

Yellow amorphous powder; ^{1}H NMR (acetone-d_6, 400 MHz): δ_H 7.76 (1H, d, *J* = 2.0 Hz, H-2'), 7.61 (1H, dd, *J* = 8.4, 1.8 Hz, H-6'), 7.26 (2H, d, *J* = 8.3 Hz, H-2 & H-6), 6.87 (1H, d, *J* = 8.8 Hz, H-5'), 6.87 (2H, dd, *J* = 8.6 Hz, H-3 & H-5); (−)-ESI-MS: *m/z* 355 $[M - H]^-$.

Variegatic acid (19)

Yellow amorphous powder; ^{1}H NMR (acetone-d_6, 400 MHz): δ_H 7.74 (1H, d, *J* = 2.0 Hz, H-2'), 7.62 (1H, dd, *J* = 8.5, 2.1 Hz, H-6'), 6.89 (1H, d, *J* = 2.0 Hz, H-2), 6.86 (1H, d, *J* = 7.6 Hz, H-5'), 6.84 (1H, d, *J* = 7.3 Hz, H-5), 6.74 (1H, dd, *J* = 7.3, 2.1 Hz, H-6); (+)-ESI-MS: *m/z* 371 $[M - H]^-$.

3*β*,5*a*,9*α*-Trihydroxy-ergosta-7,22-dien-6-one (20)

White amorphous powder; $[\alpha]_D^{25}$ –20.8 (*c* 0.18, $CHCl_3$); ^{1}H NMR ($CDCl_3$, 400 MHz): δ_H 5.63 (1H, d, *J* = 1.8 Hz, H-7), 5.23 (1H, dd, *J* = 15.0, 7.5 Hz, H-23), 5.15 (1H, dd, *J* = 15.0, 8.0 Hz, H-22), 4.04 (1H, m, H-3), 1.02 (3H, d, *J* = 6.8 Hz, H_3-21), 0.99 (3H, s, H_3-19), 0.91 (3H, d, *J* = 6.8 Hz, H_3-28), 0.83 (3H, d, *J* = 6.8 Hz, H_3-27), 0.81 (3H, d, *J* = 6.7 Hz, H_3-26), 0.61 (3H, s, H_3-18); ^{13}C NMR ($CDCl_3$, 150 MHz): δ_C 197.8 (C-6), 164.3 (C-8), 135.0 (C-22), 132.5 (C-23), 119.8 (C-7), 79.7(C-5), 74.7 (C-9), 67.2 (C-3), 56.0 (C-17), 51.8 (C-14), 45.3 (C-13), 42.8 (C-24), 41.8 (C-10), 40.3 (C-20), 34.9 (C-12), 33.1 (C-25), 33.0 (C-4), 30.0 (C-2), 28.8 (C-11), 27.8 (C-16), 25.4 (C-1), 22.4 (C-15), 21.1 (C-18), 20.4 (C-27), 19.9 (C-26), 19.6 (C-21), 17.6 (C-28), 12.2 (C-19); (+)-ESI-MS: *m/z* 467 $[M + Na]^+$.

(24*S*)-3*β*,5*α*,9*α*-Trihydroxyergost-7-en-6-one (21)

White amorphous powder; $[\alpha]_D^{25}$ –8.8 (*c* 0.07, $CHCl_3$); ^{1}H NMR (C_5D_5N, 400 MHz): δ_H 8.62 (1H, s, 5-OH), 6.30 (1H, s, 9-OH), 6.27 (1H, d, *J* = 4.9 Hz, 3-OH), 5.95 (1H, d, *J* = 2.1 Hz, H-7), 4.66 (2H, m, H_2-23), 2.97 (1H, m, H-14), 2.85 (1H, m, H-1*α*), 2.85 (1H, m, H-4*α*), 2.36 (1H, dd, *J* = 14.0, 11.2 Hz, H-4*β*), 1.17 (3H, s, H_3-19), 0.96 (3H, d, *J* = 6.0 Hz , H_3-21), 0.89 (3H, d, *J* = 6.9 Hz, H_3-28), 0.83 (3H, d, *J* = 5.2 Hz, H_3-27), 0.82 (3H, d, *J* = 5.2 Hz, H_3-26), 0.63.(3H, s, H_3-18); (+)-ESI-MS: *m/z* 469 $[M + Na]^+$.

Cerevisterol (22)

White amorphous powder; $[\alpha]_D^{25}$ –13.2 (*c* 0.14, $CHCl_3$), ^{1}H NMR (DMSO-d_6, 600 MHz): δ_H 5.23 (1H, dd, *J* = 15.3, 7.9 Hz, H-22), 5.17 (1H, dd, *J* = 15.6, 7.1 Hz, H-23), 5.08 (1H, m, H-7), 4.49 (1H, d, *J* = 5.4 Hz, 6-OH), 4.22 (1H, d, *J* = 5.8 Hz, 3-OH), 3.76 (1H, m, H-3), 3.59 (1H, s, 5-OH), 3.37 (1H, br s, H-6), 0.99 (3H, d, *J* = 7.0 Hz, H_3-21), 0.90 (3H, s, H_3-19), 0.88

(3H, d, *J* = 7.2 Hz, H_3-28), 0.81 (3H, d, *J* = 7.3 Hz, H_3-27), 0.79.(3H, d, *J* = 6.9 Hz, H_3-26), 0.54 (3H, s, H_3-18); ^{13}C NMR (DMSO-d_6, 150 MHz): δ_C 139.7 (C-8), 135.4 (C-22), 131.4 (C-23), 119.4 (C-7), 74.4 (C-5), 72.1 (C-6), 66.0 (C-3), 55.3 (C-17), 54.2 (C-14), 43.0 (C-13), 42.3 (C-9), 42.0 (C-24), 40.2 (C-2), 40.0 (C-20), 39.0 (C-12), 36.6 (C-10), 32.5 (C-11), 32.5 (C-25), 31.2 (C-4), 27.8 (C-16), 22.6 (C-15), 21.3 (C-1), 21.0 (C-21), 19.8 (C-26), 19.5 (C-27), 17.7 (C-19), 17.0 (C-28), 12.1 (C-18). (+)-ESI-MS: *m/z* 453 $[M + Na]^+$.

(22*E*,24*R*)-3*β*,5*α*-Dihydroxy-6*β*-methoxy-ergosta-7,22-diene (23)

White amorphous powder; $[\alpha]_D^{25}$ –81.1 (*c* 0.06, $CHCl_3$), ^{1}H NMR ($CDCl_3$, 400 MHz): δ_H 5.42 (1H, br d, *J* = 5.0 Hz, H-7), 5.23 (1H, dd, *J* = 15.1, 7.8 Hz, H-23), 5.17 (1H, dd, *J* = 15.1, 7.8 Hz, H-22), 4.07 (1H, m, H-3), 3.39 (3H, s, 6-OCH_3), 3.17 (1H, br d, *J* = 5.2 Hz, H-6) ,1.03 (3H, d, *J* = 6.6 Hz, H_3-21), 1.00 (3H, s, H_3-19), 0.91 (3H, d, *J* = 7.0 Hz, H_3-28), 0.84 (3H, d, *J* = 6.5 Hz, H_3-27), 0.82 (3H, d, *J* = 6.5 Hz, H_3-26), 0.59 (3H, s, H_3-18); (+)-ESI-MS: *m/z* 467 $[M + Na]^+$.

(22*E*,24*R*)-Ergosta-7,9(11),22-trien-3*β*,5*α*,6*β*-triol (24)

White amorphous powder; $[\alpha]_D^{25}$ –6.8 (*c* 0.09, $CHCl_3$), ^{1}H NMR ($CDCl_3$, 400 MHz): δ_H 5.75 (1H, br d, *J* = 7.0 Hz, H-11), 5.44 (1H, br d, *J* = 5.7 Hz, H-7), 5.25 (1H, dd, *J* = 15.5, 7.8 Hz, H-23), 5.16 (1H, dd, *J* = 15.3, 7.8 Hz, H-22), 4.12 (1H, m, H-3), 3.81 (1H, brs, H-6), 1.28 (3H, s, H_3-19), 1.02 (3H, d, *J* = 6.7 Hz, H_3-21), 0.92 (3H, d, *J* = 6.8 Hz, H_3-28), 0.84 (3H, d, *J* = 6.7 Hz, H_3-27), 0.82 (3H, d, *J* = 6.7 Hz, H_3-26), 0.60 (3H, s, H_3-18). (+)-ESI-MS: *m/z* 451 $[M + Na]^+$.

(22*E*,24*R*)-5*α*,6*α*-Epoxy-ergosta-8,22-dien-3*β*,7*β*-diol (25)

White amorphous powder; $[\alpha]_D^{25}$ –10.9 (*c* 0.11, $CHCl_3$), ^{1}H NMR ($CDCl_3$, 400 MHz): δ_H 5.22 (1H, dd, *J* = 15.8, 7.6 Hz, H-23), 5.16 (1H, dd, *J* = 15.8, 7.6 Hz, H-22), 4.38 (1H, br s, H-7), 3.93 (1H, m, H-3), 3.14 (1H, br d, *J* = 2.8 Hz, H-6), 2.20 (1H, dd, *J* = 12.0, 11.6 Hz, H-4*β*), 1.28 (3H, s, H_3-19), 1.02 (3H, d, *J* = 6.4 Hz, H_3-21), 0.91 (3H, d, *J* = 6.7 Hz, H_3-28), 0.83 (3H, d, *J* = 6.5 Hz , H_3-27), 0.82 (3H, d, *J* = 6.5 Hz, H_3-26), 0.63 (3H, s, H_3-19); (+)-ESI-MS: *m/z* 393 $[M – 2H_2O + H]^+$.

(22*E*,24*R*)-5*α*,6*α*-Epoxy-ergosta-8,22-dien-3*β*,7*α*-diol (26)

White amorphous powder; $[\alpha]_D^{25}$ –23.5 (*c* 0.07, $CHCl_3$), ^{1}H NMR ($CDCl_3$, 400 MHz): δ_H 5.20 (1H, dd, *J* = 15.4, 7.5 Hz, H-23), 5.17 (1H, dd, *J* = 15.4, 7.5 Hz, H-22), 4.23 (1H, br d, *J* = 10.8 Hz, H-7), 3.96 (1H, m, H-3), 3.31 (1H, d, *J* = 2.6 Hz, H-6), 1.26 (1H, d, *J* = 10.8 Hz, 7-OH), 1.14 (3H, s, H_3-19), 1.02 (3H, d, *J* = 6.8 Hz, H_3-21), 0.91 (3H, d, *J* = 6.6 Hz, H_3-28), 0.83 (3H, d, *J* = 7.0 Hz, H_3-27), 0.81 (3H, d, *J* = 7.0 Hz, H_3-26), 0.59 (3H, s, H_3-18); (+)-ESI-MS: *m/z* 451 $[M + Na]^+$.

(22*E*,24*R*)-5*α*,6*α*-Epoxy-ergosta-8(14),22-dien-3*β*,7*α*-diol (27)

White amorphous powder; $[\alpha]_D^{25}$ –26.7 (*c* 0.06, $CHCl_3$), ^{1}H NMR ($CDCl_3$, 400 MHz): δ_H 5.21 (1H, dd, *J* = 15.7, 7.4 Hz, H-23), 5.19 (1H, dd, *J* = 15.8, 7.4 Hz, H-22), 4.40 (1H, br s, H-7), 3.91 (1H, m, H-3), 3.14 (1H, br d, *J* = 3.5 Hz, H-6), 1.01 (3H, d, *J* = 6.8 Hz, H_3-21), 0.91 (3H, d, *J* = 6.8 Hz, H_3-28), 0.86 (6H, s, H_3-18 & H_3-19), 0.83 (3H, d, *J* = 6.5 Hz, H_3-26), 0.82 (3H, d, *J* = 6.5 Hz, H_3-27); (+)-ESI-MS: *m/z* 451 $[M + Na]^+$.

(22*E*,24*R*)-5*α*,6*α*-Epoxy-7*α*-methoxy-ergosta-8(14),22-dien-3*β*-ol (28)

White amorphous powder; $[\alpha]_D^{25}$ –31.3 (*c* 0.06, $CHCl_3$), ^{1}H NMR ($CDCl_3$, 400 MHz): δ_H 5.23 (1H, dd, *J* = 15.5, 6.5 Hz, H-23), 5.16 (1H, dd, *J* = 15.5, 6.5 Hz, H-22), 4.16 (1H, d, *J* = 3.2 Hz, H-7), 3.93 (1H, m, H-3), 3.41 (3H, s, 7-OMe), 3.20 (1H, br d, *J* = 3.2 Hz, H-6), 1.01 (3H, d, *J* = 6.7 Hz, H_3-21), 0.91 (3H, d, *J* = 6.8 Hz, H_3-28), 0.86 (6H, s, H_3-18 & H_3-19), 0.83 (3H, d, *J* = 6.7 Hz, H_3-26), 0.82 (3H, d, *J* = 6.5 Hz, H_3-27). (+)-ESI-MS: *m/z* 465 $[M + Na]^+$.

(22*E*,24*R*)-5*α*,6*α*-Epoxy-3*β*-hydroxy-ergosta-8,22-dien-7-one (29)

White amorphous powder; $[\alpha]_D^{25}$ +11.2 (*c* 0.13, $CHCl_3$), ^{1}H NMR ($CDCl_3$, 400 MHz): δ_H 5.22 (1H, dd, *J* = 15.6, 8.2 Hz, H-23), 5.15 (1H, dd, *J* = 15.6, 8.2 Hz, H-22), 3.98 (1H, m, H-3), 3.29 (1H, s, H-6), 1.25 (3H, s, H_3-19), 1.02 (3H, d, *J* = 6.6 Hz, H_3-21), 0.92 (3H, d, *J* = 7.8 Hz, H_3-28), 0.84 (3H, d, *J* = 6.6 Hz, H_3-27), 0.82 (3H, d, *J* = 6.6 Hz, H_3-26), 0.59 (3H, s, H_3-18); ^{13}C NMR ($CDCl_3$, 150 MHz): δ_C 196.2 (C-7), 157.9 (C-9), 135.3 (C-22), 132.2 (C-23), 128.8 (C-8), 68.6 (C-3), 64.5 (C-5), 62.5 (C-6), 53.3 (C-17), 48.9 (C-14), 42.8 (C-24), 42.1 (C-13), 40.5 (C-10), 40.4 (C-20), 38.3 (C-4), 35.7 (C-12), 33.1 (C-25), 30.6 (C-2), 30.4 (C-1), 29.5 (C-16), 25.2 (C-11), 24.3 (C-15), 24.1 (C-19), 21.1 (C-21), 19.9 (C-27), 19.6 (C-26), 17.6 (C-28), 11.6 (C-18). (+)-ESI-MS: *m/z* 449 $[M + Na]^+$.

(22*E*,24*R*)-3*β*,5*α*-Dihydroxy-ergosta-7,22-dien-6,5-olide (30)

White amorphous powder; $[\alpha]_D^{25}$ +8.9 (*c* 0.12, MeOH); ^{1}H NMR ($CDCl_3$, 600 MHz): δ_H 5.72 (1H, s, H-7), 5.25 (1H, dd, *J* = 15.2, 7.5 Hz, H-23), 5.15 (1H, dd, *J* = 15.2, 8.1 Hz, H-22), 3.81 (1H, m, H-3), 3.38 (H, br s, 5-OH), 1.07 (3H, s, H_3-19), 1.02 (3H, d, *J* = 6.6 Hz, H_3-21), 0.92 (3H, d, *J* = 6.8 Hz, H_3-28), 0.85 (3H, d, *J* = 6.7 Hz, H_3-27), 0.83 (3H, d, *J* = 6.6 Hz, H_3-26), 0.63 (3H, s, H_3-18); ^{13}C NMR ($CDCl_3$, 150 MHz): δ_C 166.3 (C-6), 159.1 (C-8), 135.0 (C-22), 132.6 (C-23), 115.3 (C-7), 104.1 (C-5), 67.6 (C-3), 58.0 (C-14), 56.4 (C-17), 51.7 (C-9), 46.6 (C-13), 46.2 (C-4), 42.8 (C-10), 42.8 (C-24), 40.3 (C-20), 39.9 (C-12), 35.9 (C-1), 33.0 (C-25), 30.4 (C-2), 27.7 (C-16), 25.3 (C-11), 23.0 (C-15), 21.0 (C-21), 19.9 (C-27), 19.6 (C-26), 17.7 (C-19), 17.6 (C-28), 12.5 (C-18). (+)-ESI-MS: *m/z* 445 $[M + H]^+$.

9(11)-DHEP (= 9,11-dehydro-ergosterol peroxide, 31)

White amorphous powder; $[\alpha]_D^{25}$ –31.1 (*c* 0.12, $CHCl_3$); ^{1}H NMR ($CDCl_3$, 400 MHz): δ_H

6.60 (1H, d, *J* = 8.6 Hz, H-6), 6.29 (1H, d, *J* = 8.6 Hz, H-7), 5.43 (1H, dd, *J* = 6.1, 1.8 Hz, H-11), 5.25 (1H, dd, *J* = 15.2, 7.5 Hz, H-23), 5.16 (1H, dd, *J* = 15.2, 7.9 Hz, H-22), 4.02 (1H, m, H-3), 1.09 (3H, s, H_3-19), 1.01 (3H, d, *J* = 6.5 Hz, H_3-21), 0.92 (3H, d, *J* = 7.0 Hz, H_3-28), 0.84 (3H, d, *J* = 6.6 Hz, H_3-26), 0.83 (3H, d, *J* = 6.6 Hz, H_3-27), 0.74 (3H, s, H_3-18); (+) ESI-MS: *m/z* 409 $[M - H_2O + H]^+$.

(22*E*,24*R*)-5*α*,8*α*-Epidioxyergosta-6,22-dien-3-ol (32)

White amorphous powder; $[\alpha]_D^{25}$ –20.1 (*c* 0.12, $CHCl_3$); ^1H NMR ($CDCl_3$, 400 MHz): δ_H 6.51 (1H, d, *J* = 8.4 Hz, H-7), 6.25 (1H, d, *J* = 8.6 Hz, H-6), 5.23 (1H, dd, *J* = 15.3, 7.4 Hz, H-22), 5.15 (1H, dd, *J* = 15.7, 8.1 Hz, H-23), 3.97 (1H, m, H-3), 1.00 (3H, d, *J* = 6.8 Hz, H_3-21), 0.91 (3H, d, *J* = 6.9 Hz, H_3-28), 0.89 (3H, s, H_3-19), 0.84 (3H, d, *J* = 6.6 Hz, H_3-26), 0.82 (3H, s, H_3-18), 0.82 (3H, d, *J* = 6.6 Hz, H_3-27); (+) ESI-MS: *m/z* 411 $[M - H_2O + H]^+$.

Demethylincisterol A_3 (33)

White amorphous powder; $[\alpha]_D^{25}$ +20.6 (*c* 0.11, $CHCl_3$), ^1H NMR ($CDCl_3$, 400 MHz): δ_H 5.64 (1H, d, *J* = 1.8 Hz, H-2), 5.26 (1H, dd, *J* = 15.2, 8.0 Hz, H-16), 5.17 (1H, dd, *J* = 15.2, 8.0 Hz, H-15), 1.04 (3H, d, *J* = 6.8 Hz, H_3-14), 0.92 (3H, d, *J* = 6.8 Hz, H_3-21), 0.84 (3H, d, *J* = 6.8 Hz, H_3-19), 0.83 (3H, d, *J* = 6.8 Hz, H_3-20), 0.61 (3H, s, H_3-12); (−) ESI-MS: *m/z* 331 $[M - H]^-$.
